# In *Porphyromonas gingivalis* VimF Is Involved in Gingipain Maturation through the Transfer of Galactose

**DOI:** 10.1371/journal.pone.0063367

**Published:** 2013-05-24

**Authors:** Arun S. Muthiah, Wilson Aruni, Antonette G. Robles, Yuetan Dou, Francis Roy, Hansel M. Fletcher

**Affiliations:** Division of Microbiology and Molecular Genetics, Department of Basic Sciences, School of Medicine, Loma Linda University, Loma Linda, California, United States of America; University of Florida, College of Dentistry & The Emerging Pathogens Institute, United States of America

## Abstract

Previously, we have reported that gingipain activity in *Porphyromonas gingivalis*, the major causative agent in adult periodontitis, is post-translationally regulated by the unique Vim proteins including VimF, a putative glycosyltransferase. To further characterize VimF, an isogenic mutant defective in this gene in a different *P. gingivalis* genetic background was evaluated. In addition, the recombinant VimF protein was used to further confirm its glycosyltransferase function. The *vimF*-defective mutant (FLL476) in the *P. gingivalis* ATCC 33277 genetic background showed a phenotype similar to that of the *vimF*-defective mutant (FLL95) in the *P. gingivalis* W83 genetic background. While hemagglutination was not detected and autoaggregation was reduced, biofilm formation was increased in FLL476. HeLa cells incubated with *P. gingivalis* FLL95 and FLL476 showed a 45% decrease in their invasive capacity. Antibodies raised against the recombinant VimF protein in *E. coli* immunoreacted only with the deglycosylated native VimF protein from *P. gingivalis*. *In vitro* glycosyltransferase activity for rVimF was observed using UDP-galactose and *N*-acetylglucosamine as donor and acceptor substrates, respectively. In the presence of rVimF and UDP-galactose, a 60 kDa protein from the extracellular fraction of FLL95 which was identified by mass spectrometry as Rgp gingipain, immunoreacted with the glycan specific mAb 1B5 antibody. Taken together, these results suggest the VimF glycoprotein is a galactosyltransferase that may be specific for gingipain glycosylation. Moreover, galatose is vital for the growing glycan chain.

## Introduction

*Porphyromonas gingivalis*, a Gram-negative, anaerobic bacterium, is a major etiological agent implicated in adult periodontal disease and is associated with other systemic diseases, including cardiovascular disease [1]–[5]. This data, taken together, implies a significant impact of this organism on the overall health of humans. The ability of this asaccharolytic bacterium to produce proteases has been shown to contribute significantly toward its pathogenicity [6]. A key element in modulating the pathogenic potential of *P. gingivalis* is the post-translational modification of the major proteases, called gingipains [7]. These consist of arginine-specific (Arg-gingipain [Rgp]) and lysine-specific (Lys-gingipain [Kgp]) cysteine proteases that are both extracellular and cell membrane associated [8]. The maturation pathway of the gingipains including its secretion facilitated by a novel POR secretion system (PorSS) is linked to carbohydrate biosynthesis. This pathway is regulated by several proteins including the PorR, PorT, Sov, Rfa, VimA, VimE, VimF and other components of PorSS [Reviewed in [9]–[11]]. However, there still remains a gap in our comprehensive understanding of the glycosylation process important in gingipain biogenesis. More specifically, the role of VimF in this process is still unclear.

The *bcp-recA-vimA-vimE-vimF-aroG* operon is essential for the maturation/activation/anchorage of the gingipains and regulation of other virulence factors of *P. gingivalis* [10]. Previously, we have reported that the *vimF* gene can affect the phenotypic expression and distribution of the gingipains in *P. gingivalis* [12]. Using the cloned *vimF* gene, a defective mutant was constructed by allelic exchange in W83. This isogenic mutant designated *P. gingivalis* FLL95, when plated on Brucella blood agar was non-pigmented and non-hemolytic. In contrast to the parent strain, arginine- and lysine-specific gingipain activities were reduced by approximately 97% and 96%, respectively. These activities were unaffected by the growth phase in contrast to the *vimA*-defective mutant *P. gingivalis* FLL92. Expression of the *rgpA*, *rgpB* and *kgp* gingipain genes were unaffected in *P. gingivalis* FLL95 when compared to the wild-type strain. In non-active gingipain extracellular protein fractions, multiple high molecular weight proteins immunoreacted with gingipain specific antibodies. However, the specific phosphorylated mannan oligosaccharide moiety recognized by the monoclonal antibody 1B5 [13] was absent in gingipains from FLL95. Taken together, these results suggest that the VimF protein which is a putative glycosyltransferase group 1 is involved in the regulation of gingipain biogenesis in *P. gingivalis* through glycosylation.

Glycosyltransferases (GTases) catalyze the transfer of monosaccharide or oligosaccharides primarily from an activated sugar donor (UDP sugars) to various substrates, including carbohydrates, proteins and glycoproteins [14]. Their physiologic significance is further highlighted by the fact that they, along with glycosidases, make up 1 to 2% of the encoded genes in living organisms [15]. Recently, various reports have associated glycosyltransferases with the biogenesis of several virulence components of *P. gingivalis* like capsule [16], fimbriae [17], lipopolysaccharide [18] and gingipains [12]. The carbohydrate composition of the gingipains which is estimated to be 14% to 30% by weight underscores the importance of glycosylation in their maturation process [13].

The post-translational addition of carbohydrates to the gingipains is highly variable, thus implying a role for multiple factors in this process [11], [13]. The attachment of carbohydrates to proteins can be either *N*- and/or *O-*linked. The *N*-linked attachment is to the amide nitrogen of asparagine facilitated by a consensus amino acid sequence of Asn-X-Ser/Thr (N-X-S/T), where X is any amino acid except proline [19]. The O-glycosidic linkage occurs via glycan attachment to the hydroxyl group of serine (S), or threonine (T) [19]. These attachments of sugar to the amino acid chain and glycans on glycoproteins are both mediated by glycosyltransferses. While several GTases are present in the genome of *P. gingivalis* [20] their specific effect on gingipain maturation is less clear. In this report, we have further characterized the putative glycosyltransferase VimF and demonstrated its ability as a galactosyltransferase involved in glycosylation of the pro-gingipain species in *P. gingivalis*.

## Materials and Methods

### Bacterial Growth Conditions and Gingipain Assays

All strains of *P. gingivalis* were grown in brain heart infusion (BHI) broth (Difco Laboratories, Detroit, MI) supplemented with hemin (5 µg/ml), vitamin K (0.5 µg/ml) and cysteine (0.1%). Defibrinated sheep blood (5%) and agar (10%) were used in blood agar plates. *Escherichia coli* strains were grown in Luria-Bertani (LB) broth. Unless otherwise stated, all cultures were incubated at 37°C. *P. gingivalis* strains were maintained in an anaerobic chamber (Coy Manufacturing, Ann Arbor, MI) in 10% H_2_, 10% CO_2_, and 80% N_2_. Growth rates for *P. gingivalis* and *E. coli* strains were determined spectrophotometrically (optical density at 600 nm [OD_600_]). Antibiotics were used at the following concentrations: clindamycin, 0.5 µg/ml; erythromycin, 300 µg/ml; and carbenicillin, 50 to 100 µg/ml. Rgp and Kgp activities were determined using the microplate reader (Bio-Rad Laboratories, Hercules, CA) as previously reported [21].

### DNA Isolation, Analysis and Cloning of the *vimF* Gene

Chromosomal DNA was extracted from *P. gingivalis* W83, 33277 and isogenic mutants (Table 1) as previously described [22]. Alkaline lysis method was used for plasmid DNA extraction [23]. Electrophoresis of DNA was done using 0.8% agarose gel prepared in TAE buffer as reported elsewhere [12]. The pTrcHis2-TOPO TA expression vector (Invitrogen, Carlsbad, CA) was used for generating the rVimF protein. Briefly, the 1.2-kb *vimF* open reading frame without stop codon was amplified from *P. gingivalis* W83 chromosomal DNA using P1 and P2 oligonucleotide primers (Table 2). The amplified fragment was purified using the QIAquick PCR Purification kit (Qiagen, Valencia, CA) then cloned into the pTrcHis2 plasmid vector following the manufacturer’s protocol. This recombinant plasmid was then used to transform *E. coli* Top 10 competent cells that were then plated on LB agar containing 50 µg/ml of ampicillin. Recombinant plasmids, named pFLL477 (Table 1), isolated from several ampicillin resistant colonies were screened for the correct orientation of the insert using PCR and confirmed by digestion with *Kpn*I and *Sph*I. One randomly chosen ampicillin resistant transformant carrying the recombinant plasmid pFLL477 was chosen for further studies. DNA sequencing was used to confirm the absence of any mutation in the *vimF* ORF.

**Table 1:** Plasmid and bacterial strains used in this study.

Plasmid/Strains	Phenotype and description	Reference
Plasmid		
pTrcHis2 TOPO	*amp^r^*, *lacI^q^*	Invitrogen
pFLL477	pTrcHis2 TOPO containing the *vimF* gene	This study
Bacterial Strains		
*P. gingivalis*		
W83	Wild type	(Abaibou, 2001)
FLL95	*vimF* mutant in W83	(Vanterpool, 2005)
FLL95C’	Complemented FLL95	This study
ATCC 33277	Wild type	
FLL476	*vimF* mutant in ATCC 33277	This study
FLL476C’	Complemented FLL476	This study
*E. coli*		
DH5α	F^-^Φ80d*lacZ*Δ M15 Δ(*lacZYA*-argF) U169 *recA1endA1 hsdR17* (r_k_^-^, m_k_^+^)*phoA supE44* λ^-^ *thi-1 gyrA96 relA1*	Invitrogen
Top10	F^-^ *mcrA* Δ(*mrr-hsdRMS-mcrBC)* Φ80d*lacZ*Δ M15 Δ*lacX74 recA1 ara139*Δ(ara-leu)7697 *galU galK rpsL* (StrR) *endA1 nupG*	Invitrogen

**Table 2:** Primers used in this study.

Primer	Description	Sequence
P1	*vimF* forward	5′-ATGAAACGGGTACTCATCTTCGCCGA-3′
P2	*vimF* reverse	5′-GTTAGCGACGATCGATTCCAGTAGAC-3′
P3	*vimF* 1 Kb upstream	5′CGGGAAGAGAGTCCTTGCTTTTCAAAGCA-3′
P4	*vimF-erm* reverse	5′-GTCATTTATTCCTCCTAGTTAGTCATGGTCGATGG CCGTTTCGTAGTCG-3′
P5	*vimF-erm* forward	5′-TTCGTAGTACCTGGAGGGAATAATCATTCAGCAT CGTATCATGAAGTAC-3′
P6	*vimF* 1 Kb downstream	5′-CTG CAG TAC GGG CAC GGT TG-3′
P7	*erm_F* forward	5′-TGACTAACTAGGAGGAATAAATGACAAAAAAGAAATTGCCCG-3′
P8	*erm_F* reverse	5′-GATTATTCCCTCCAGGTACTACGAAGGATGAAATTTTTCA-3′
P9	*vimF* complement forward	5′-GAT CGG AAA GCA GCG CAA GCG ACT TAT-3′
P10	*vimF* complement reverse	5′-ATC TGT CGA ACT CCG GAC TGC CG-3′

### Purification of rVimF

An overnight culture of the *E. coli* Top 10 cells carrying pFLL477 was used to inoculate two liters of prewarmed LB broth containing 50 µg/ml ampicillin. The culture was then grown at 37°C to the exponential phase (OD_600 = _0.6) after which it was induced with 1 mM IPTG and further incubated for 5 hours. The cells were harvested by centrifugation (2,400 g for 20 minutes) and washed twice with 10 mM Tris-HCl at pH 7.4. The cell pellet was suspended in binding buffer (20 mM NaH_2_PO4, 500 mM NaCl and 40 mM Imidazole) and frozen at −20°C. The cells were thawed and lysed by French pressure cell press with five passes in the presence of Mini EDTA-free protease inhibitor tablets (Roche, Indianapolis, IN) after the first and the last passes. After centrifuging the lysate at 2,400 g for 20 minutes to remove cell debris, the cleared supernatant was further centrifuged at 100,000 g for 1 h. The resultant supernatant was either stored at −80°C and used in a glycosyltransferase assay or, mixed with 1 liter of binding buffer containing 0.5% tween and loaded on to the His-Prep FF 16/10 column (GE Healthcare, Piscataway, NJ) for protein purification. After washing the column twice with two column volumes of wash buffer (same as binding buffer), the bound proteins were eluted with buffer containing 500 mM imidazole, 20 mM NaH_2_PO4 and 500 mM NaCl. Fractions containing the 50 kDa proteins were pooled, buffer exchanged with 10 mM Tris-HCl (pH 7.4) using 10,000 MW cutoff membrane in an ultrafiltration cell (Amicon Inc., Beverly, MA) and concentrated using a speed vacuum concentrator (Savant Instrument, Inc., Farmingdale, NY).

### Production of Rabbit Polyclonal Antibodies against the rVimF Protein

To avoid the 60 kDa GroEL band that was observed to co-purify with rVimF, the purified rVimF (25 µg/lane) was separated by SDS-PAGE using NuPAGE 4 to 12% Bis-Tris gels and excised for antibody production. A total of approximately 1.2 mg of the rVimF protein was excised from the gels, placed in 1× PBS buffer, and sent to Open Biosystems Inc., Huntsville, AL., for the production of polyclonal rabbit VimF antibodies by using the manufacturer’s standard protocol. Dilutions and efficiency of the antibodies were tested in the laboratory with the purified rVimF. All serum was aliquoted and stored at −80°C.

### Preparation of *P. gingivalis* Total Cell and Cell Free Supernatant Fractions

Total cell lysate and extracellular fractions were collected from *P. gingivalis* W83 and FLL95. Cells were grown to log phase and centrifuged at 10,000 g for 30 minutes at 4°C. The proteins from the cell-free supernatant were precipitated with ammonium sulphate (80%). The protein pellet was re-suspended in 10 mM Tris-HCl (pH 7.4) and dialyzed extensively against the same buffer to remove ammonium sulphate. The cell pellet was washed two times with 10 mM Tris-HCl (pH 7.4) and kept at −20°C. The cells were lysed by French Pressure Cell Press (American Instrument Company, Silver Spring, MD) as previously described [12]. Following centrifugation for 10,000 g for 30 minutes to remove cell debris, the supernatant was designated as the total cell lysate.

### Purification of Gingipain Protease

The gingipains were purified as previously reported [24] with some modifications. Ammonium sulfate instead of acetone precipitation was used to precipitate the gingipains from the culture supernatant of *P. gingivalis* FLL95 or W83 grown to OD_600_ of 0.8–1.0. In addition, four columns were used in the following order: Hi Load 16/60 Superdex 200 (GE Healthcare, Piscataway, NJ), DEAE Sepharose FF XK16 anion exchange column (Amersham Bioscience, Piscataway, NJ), Arginine Sepharose column (GE Healthcare, Piscataway, NJ), followed by the Superdex 200 HR 10/30 column (Amersham Bioscience, Piscataway, NJ).

### SDS-PAGE and Western Blotting

10% SDS-PAGE gel was used for protein separation of purified rVimF and cell lysates of *E.coli* and *P. gingivalis* strains. Samples were mixed with approximately 10% NuPAGE reducing agent and 25% 4X LDS buffer and heated for 10 minutes at 72°C. Electrophoresis was done at 130 V for 70 minutes and stained with SimplyBlue SafeStain for visualization. Nitrocellulose membrane with pore size 0.45 µm (Schleicher & Schuell, Reviera Beach. FL) was used for blotting using 15 V for 25 min in a Semi-Dry Trans-blot apparatus (Bio-Rad, Hercules, CA). These blots were probed by using either rabbit anti-rVimF antibody (1 in 4000 dilution) or, mouse mAb IB5 (1 in 20 dilution) demonstrated to immunoreact with gingipain-associated sugar moiety [13]. Primary antibody was allowed to react with the membrane for 1 hour and, following 4 washing steps, secondary antibody (HRP conjugated goat anti-rabbit or goat anti-mouse, both in 1 in 4000 dilution) was allowed to react for 30 minutes. Following 2 more washing steps, immunoreactive proteins were detected by the procedure described in the Western Lightning Chemiluminescence Reagent Plus kit (Perkin-Elmer Life Sciences, Boston, MA).

### Glycosyltransferase Assay

#### Calibration curve

A calibration curve, as previously described [25], was generated to establish the relationship between proton production and change in absorbance of the pH indicator. The reaction mixture (1 ml final volume) contained 2 mM sodium phosphate buffer (pH 8), 0.01 mM phenol red, 0.1 mM MnCl2, 10 mM *N*- acetylglucosamine, 100 µl of *E. coli* Top 10 cells expressing pFLL477 lysate and different volumes of HCl (10 mM) was added to get final concentrations of 0.1, 0.2, 0.3, 0.4, 0.5, 0.6, 0.7, and 0.8 mM. The absorbance of the mixture was determined spectrophotometrically (optical density at 557 nm [OD_557_]). The data points were plotted using GraphPad Prizm 5 software (La Jolla, CA).

To screen for donor and acceptor substrate, 2 mM phosphate buffer (pH 8) containing 0.1 mM phenol red, 0.1 mM MnCl_2_, 10 mM *N*-acetylglucosamine (acceptor), 100 µl of crude lysate of *E. coli* Top 10 cells containing pFLL477 and UDP-sugars (galactose or glucose) were added to a final concentration of 2 mM. The absorbance at 557 nm was monitored for each sample at 15 s intervals for a total of 60 minutes using the spectrophotometer Beckman DU 650(Beckman Coulter, Brea, CA). Other acceptor substrates used in place of *N*-acetylglucosamine were – glucose, galactose, lactose, *N*- acetylgalactosamine and mannose. All reactions were carried out at a constant temperature of 37°C. Enzyme activity was calculated using the GraphPad Prizm 5 enzyme kinetics option by intrapolating the OD_557_ values from calibration curve. A commercially available bovine β-1, 4 galactosyltransferase (Sigma, St. Louis, MO) was used as positive control and a non-specific *E. coli* Top 10 cell lysate served as negative control. All enzymatic assays were done in triplicate and values averaged.

### *P. gingivalis* Proteins as GTase Acceptor Substrate

Extracellular and whole cell lysates of W83 and FLL95 were used as acceptors in the *in-vitro* Galactosyl transferase assay [25] in the presence of UDP-galactose (donor). Briefly, in a total reaction volume of 16 µl, supernatant containing about 7 to 15 µg of *P. gingivalis* extracellular protein (in 10 mM Tris-HCl) was mixed with 5 µg of *E. coli* lysate (containing pFLL477 producing the rVimF protein) and 1 µl of 0.8 mM UDP-galactose. This mixture was incubated at 37°C for 2 hours. Similar reactions omitting rVimF lysate and/or UDP-galactose served as controls. After incubation, the reaction was stopped by adding 4X lithium dodecyl sulphate (LDS) buffer (Invitrogen, Carlsbad, CA), reducing agent and water to make up a final volume of 20 µl. The samples were denatured at 72°C for 10 minutes then separated on 10% SDS-PAGE at 130 V for 70 minutes. Carbohydrate specific modifications were visualized using the glycan specific mAb 1B5 antibody in western blot analysis as described elsewhere [13].

### Glycoprotein Staining of rVimF and Gingipains

Purified rVimF along with positive and negative controls provided in the Glycoprotein Staining Kit (Pierce, Rockford, IL) were separated by SDS-PAGE and then transferred to nitrocellulose membrane and stained as per manufacturer’s instructions. The membrane was stored in deionized water. Glycoproteins were seen as magenta bands with light pink or colorless background. For glycoprotein staining of gingipains, equivalent amounts of purified proteins (W83 catalytic domain and FLL95 proenzyme) were resolved on a 10% separating gel using sodium dodecyl sulfate-polyacrylamide gel electrophoresis (SDS-PAGE) in MOPS (Morpholinepropanesulfonic acid)-SDS running buffer according to manufacturer’s instructions. Glycoprotein stain was then performed on the gel using Pierce Glycoprotein Staining Kit as per manufacturer’s instructions. An equivalent gel was stained using SimplyBlue SafeStain (Invitrogen, Carlsbad, CA) for comparison.

### Determination of Glycosyl Composition of Proenzyme from FLL95

Protein samples (100 µg) were dried and the monosaccharide composition of the proenzyme from FLL95 was determined by methanolysis and silylation followed by GC-MS analysis of trimethylsilyl (TMS)-methyl glycosides [26], with the addition of a reacetylation step just prior to silylation using 25 µl of methanol, 25 µl of pyridine, and 25 µl of acetic anhydride at room temperature for 15 minutes, in order to detect amino sugars.

### Tryptic Digestion and Mass Spectroscopy

SDS-PAGE separated protein bands and spots from 2D gels were excised and subjected to digestion with trypsin. The gel slices were first transferred to low retention epi vials (Fisher, Hampton, NH) and dehydrated using neat acetonitrile for 30 minutes. 20 µl of TCEP (tris(2-carboxyethyl)phosphine) was then added and incubated for 1 hr. at 60°C. In the next step, 40 µl alkylating buffer (200 mM iodoacetamide) was added and incubated at room temperature for one hour. The gel slice was washed in 0.5 ml of neat acetonitrile and re-suspended in another 0.5 ml of neat acetonitrile to dehydrate. Next, digestion buffer containing mass spectroscopy grade trypsin in 50 mM NH_4_HCO_3_ was added to attain a 1∶20 to 1∶50 enzyme/substrate ratio and incubated overnight at 40°C. Digestion was stopped using 10 µl of 10% formic acid. Digested peptides were extracted using standard C_18_ Zip Tip technology (Millipore, Bedford, MA) according to manufacturer’s protocol. MS analysis of extracted peptide was done as described elsewhere [27].

### Inactivation of the *vimF* Gene in *P. gingivalis* ATCC 33277

Fusion PCR, used successfully to inactivate genes in our lab [28], [29], was used to inactivate the *vimF* gene in 33277. Briefly, a 1 Kb region upstream of *vimF* was amplified with a 5′ overhang that was complementary to a 3′ region of *ermF* using primers P3 and P4 (Table 2) and, 1 Kb downstream of *vimF* was amplified with 3′ overhang complementary to 5′ end of *ermF* by using primers P5 and P6 (Table 2). *ermF* was amplified separately using primer P7 and P8 (Table 2). Finally, the purified upstream, downstream and *ermF* fragments were combined in one PCR reaction using primer P3 and P6 to replace *vimF* by *ermF* by PCR. The fused fragment was purified and electroporated into *P. gingivalis* 33277 cells. The electrotransformed cells were plated on BHI blood agar plate containing 10 µg/ml of erythromycin and incubated for 8–10 days. Non-black pigmented colonies on blood agar were screened for the correct gene replacement by PCR and DNA sequencing. One isogenic mutant randomly chosen and designated FLL476 (33277Δ*vimF*) was used for further studies.

### Complementation of *vimF* Mutants

PCR mediated gene replacement was used to complement the *vimF* defective mutants. Briefly, using primers P9 and P10 (Table 2) the ORF of *vimF* with 500 bp flanking regions of both upstream and downstream was first amplified from *P. gingivalis* W83 and 33277 chromosomal DNA and purified using the QIAEX Gel Extraction Kit (Qiagen, Valencia, CA). This purified fragment was electroporated into *P. gingivalis* FLL95 or FLL476 cells grown to exponential phase (OD_600_ = 0.6). Electroporated cells were incubated for 12 hours in 1 ml of BHI broth then plated on BHI blood agar plates. Plates were then screened after 8 days for black pigmented colonies. These colonies were subsequently checked for the presence of the uninterrupted *vimF* gene. One randomly chosen colony designated *P. gingivalis* FLL95C’ or FLL476C’ was chosen for further study.

### Autoaggregation, Hemagglutination and Biofilm Assays

Autoaggregation assays of *P. gingivalis* ATCC 33277 and FLL476 was performed as previously described [30] with slight modification. Briefly, *P. gingivalis* cells in the early to mid-log phase was collected by centrifugation, washed three times with PBS, and then re-suspended in PBS to an OD_600_ of 1.0. Autoaggregation was monitored by the decrease in OD_600_ of each suspension over a three hour period at 37°C.

Hemagglutination activity was determined as previously reported [31]. After serially diluting the bacterial suspension in a round bottom 96-well microtiter plates an equal volume of (100 µl) of 1% PBS washed sheep erythrocytes was mixed with each dilution and incubated at 4°C for 3 h. Hemagglutination was visually assessed and the last dilution showing complete hemagglutination was taken as the titer.

Biofilm formation was estimated as previously described [32] with little modification. Briefly, *P. gingivalis* cells grown overnight was washed twice with 1× PBS buffer and re-suspended in BHI-PBS (ratio 1∶2) at OD_600_ of 0.2–0.3. 100 µl of this cell suspension was added to multiple wells of a pre-sterilized 96 well plate, covered and incubated overnight at 37°C anaerobically. Next day, free floating cells were aspirated and wells were washed four times with 100 µl of 1× PBS. After drying the plates at 37^ o^C for 30 minutes, 100 µl of 0.5% (w/v) crystal violet was added to the wells and incubated for 30 minutes at room temperature. After removing the crystal violet solution, the wells were washed four times with 1× PBS and de-stained using 100 µl of 95% ethanol for 30 minutes. The released crystal violet was collected in a cuvette and after adding 500 µl of ddH_2_O the biofilm formation was measured for each well at OD_595_.

### Electron Microscopic Analysis

Tecnai G2 20 Transmission Electron Microscope was used as previously reported [33], to visualize the surface structure of wild type *P. gingivalis* W83 and ATCC 33277 strains compared with their corresponding respective isogenic mutants FLL95 and FLL476, respectively. Briefly, Formvar-carbon coated grids were prepared; the Formvar support was removed by placing the grids in an atmosphere of solvent vapor. Grids were then placed on a wire mesh in a glass Petri dish, with carbon tetrachloride below the wire mesh. Cultures at OD_600_ = 0.8 was pre-clarified and washed twice with PBS (pH 7.2). The final pellet was dissolved in PBS to get OD_600_ of 0.7. About 200 µl of the processed sample was loaded into a 500 mesh. The grids were then washed in 0.5% acetic acid then by acetone. The carbon film was broken to free the specimen grid, after which the grid was placed in stain solution - neutral 1% aqueous phosphotungstic acid for 30 seconds. After blotting dry, the grid was examined using the Tecnai G2 20 Transmission Electron Microscope.

### Adherence and Antibiotic Protection Assay

The HeLa cells were grown and maintained in the Dulbecco’s modified Eagle’s medium supplemented with 10% fetal bovine serum, penicillin (100 IU/ml), streptomycin (100 IU/ml), and amphotericin B (2.5 mg/ml) (Invitrogen, Carlsbad, CA), at 37°C under 5% CO_2_ atmosphere. Confluent stock cultures were trypsinized, adjusted to approximately 5×10^3^ cells/ml, seeded into 12-well plates (Nunc, Rochester, NY) 1 ml per well and further incubated for 48 h to reach semi-confluency (10^5^ cells per well). Standard antibiotic protection assay was used to quantify invasion [34]. Briefly, an isolated colony harvested from solid agar plate was grown to exponential phase in BHI broth. The cells were centrifuged, washed three times in 1× PBS, and adjusted to 10^7^ CFU/ml of bacteria (confirmed by colony count) in Dulbecco’s modified Eagle’s medium. Epithelial cell monolayers were washed three times with PBS, infected with bacteria at a multiplicity of infection (MOI) of 1∶100 (10^5^ epithelial cells), and then incubated at 37°C for 90 min under a 5% CO_2_ atmosphere. Non-adherent bacteria were removed by washing with PBS, while cell surface bound bacteria would be killed with metronidazole (200 µg/ml, 60 min). *P. gingivalis* in general is sensitive to 100 µg/ml metronidazole. After removal of antibiotic, the internalized bacteria were released by osmotic lysis in sterile distilled water with scraping. Lysates were serially diluted, plated (in duplicate) on BHI agar, and incubated for 6 to 10 days. The number of bacterial cells recovered was expressed as percentage of the original inoculum. The number of adherent bacteria was obtained by subtracting the number of intracellular bacteria from the total number of bacteria obtained in the absence of metronidazole.

## Results

### VimF Defective Mutant Displays a Similar Phenotype in a Different Genetic Background of *P. gingivalis*

**Figure 1:**
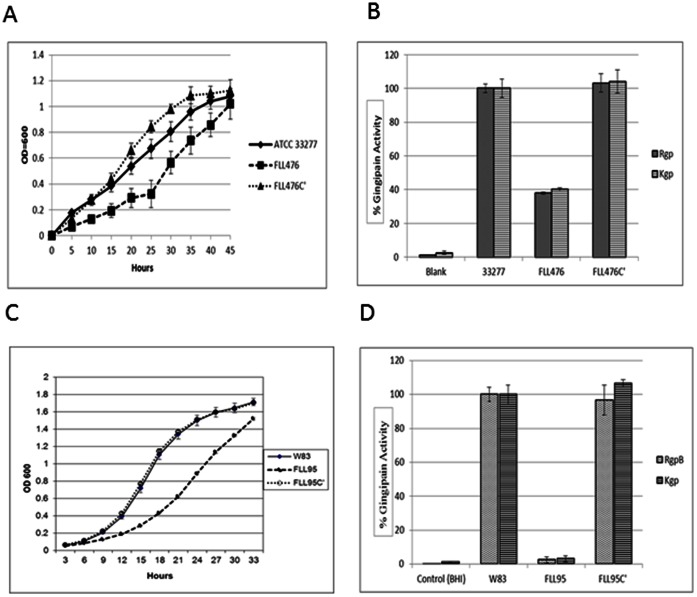
Comparison of growth and gingipain activities of wild-type, *vimF* mutant and complemented strains of W83 and ATCC 33277. Growth rate of *P. gingivalis* ATCC 33277 (**A**) and W83 (**C**) were compared with their respective *vimF*-defective isogenic mutants (FLL476 and FLL95 ) and complemented strains (FLL476C’ and FLL95C’). The data shown is an average of three independent replicates. Error bars represent the SD. Gingipain activity of W83 (**D**) and ATCC 33277 (**B**) were compared with respective mutants and complemented strains. The activities were normalized to W83 and ATCC 33277 being 100% and the mutants reported as a percentage thereof. Error bars represent SD.

Inactivation of the *vimF* gene in *P. gingivalis* W83 resulted in a non-black pigmented isogenic mutant designated *P. gingivalis* FLL95, which showed reduced levels of proteolytic, hemagglutinating and hemolytic activities [12]. To further confirm this phenotype in a different genetic background, a *vimF* deletion mutant in *P. gingivalis* ATCC 33277 was constructed by allelic exchange mutagenesis. Following electroporation and plating on selective medium, several erythromycin-resistant colonies were detected after 5–7 days of incubation. To compare their phenotypic properties with those of the wild-type 33277 strain, all mutants were plated on Brucella blood agar plates. In contrast to the wild-type, all the isogenic mutants had a non-black pigmented, non-hemolytic phenotype. PCR amplification of chromosomal DNA showed that the *vimF* gene was missing in those isogenic mutants in comparison to the wild-type. One randomly chosen mutant, designated FLL476, was chosen for further characterization. The mutation was further confirmed by DNA sequencing (data not shown). Because of the use of an *ermF* cassette lacking a transcriptional terminator, inactivation of *vimF* did not have any polar effects on the expression of its downstream genes which was confirmed using PCR analysis (data not shown). In FLL476 the growth rate (Fig. 1A) and gingipain activity (Fig. 1B) were reduced to similar levels as previously observed in *P. gingivalis* FLL95 [12]. Complementation of FLL95 and FLL476 with the wild-type gene, which was confirmed using RT-PCR (data not shown), restored growth rate and gingipain activity to both W83 (Fig. 1C and 1D) and ATCC 33277 (Fig. 1A and 1B) wild type levels.

### VimF can Modulate Biofilm Formation, Autoaggregation and Hemagglutination in *P. gingivalis* ATCC 33277

**Figure 2:**
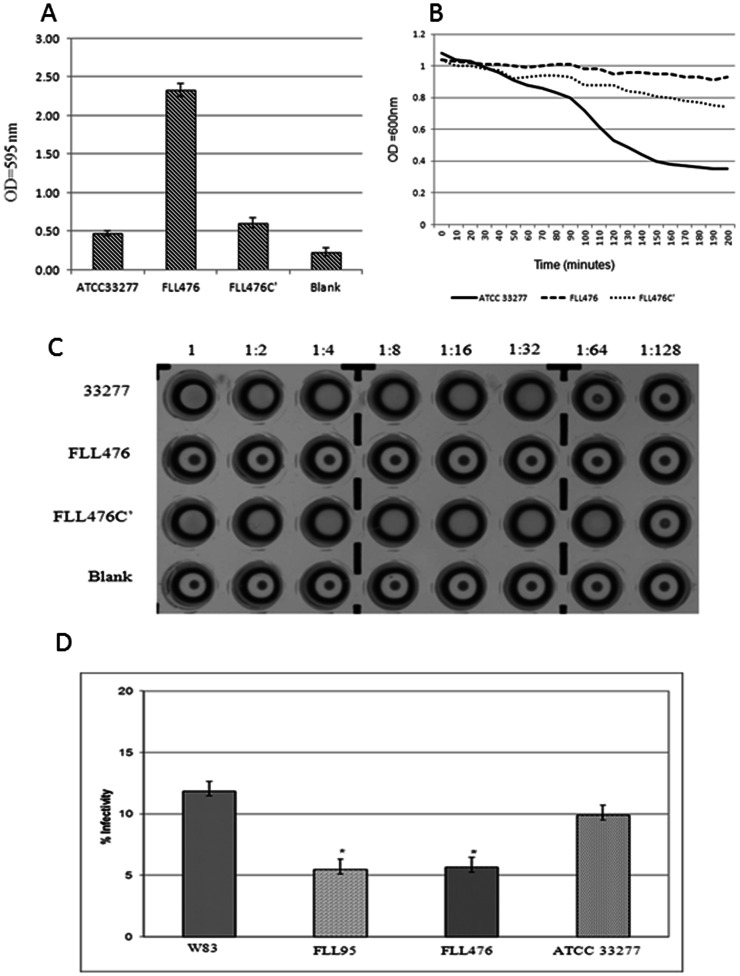
Comparison of biofilm formation, autoaggregation, hemagglutination and invasion assay. **A.** Biofilm formation of ATCC 33277, FLL476 and FLL476C’ were compared. Biofilm assay was done by staining adherent cells of overnight cultures grown in microtiter plates with 0.5% (w/v) crystal violet. Blank contained only media. Biofilm forming ability corresponded to OD_595_. **B.** Autoaggregation of 33277, FLL476 and FLL476C’ corresponded to change in OD_600_ monitored for about three hours after cells were washed and suspended in PBS. A representative sample is shown. **C.** Hemagglutination activities of ATCC 33277, FLL476 and FLL476C’ were assessed by serially diluting cells in PBS and incubating with sheep RBCs for 3 h at 4°C. Dilutions are listed above and last dilution showing matt formation was taken as the titer. The blank contained only media. **D.** Antibiotic Protection Assay was used to quantify invasion. *P. gingivalis* cells that were able to invade HeLa cell monolayers were released by lysis and cultured on BA plates. Infectivity was taken as the percentage of cells recovered. (* = p<0.05).

Alteration in *P. gingivalis* cell surface could alter their ability to autoaggregate, hemagglutinate and form biofilm [35]–[37]. To ascertain the involvement of *vimF* in cell surface modification we evaluated the ability of *vimF* mutant FLL476 to autoaggregate, hemagglutinate and form biofilm. A four-fold increase in biofilm formation was observed in FLL476 when compared to wild-type ATCC 33277 and the complemented strain FLL476C’ (Fig. 2A). Also, autoaggregation was reduced in FLL476 when compared to ATCC 33277 and FLL476C’, however the FLL476C’ did not totally regain its autoaggregation ability (Fig. 2B). As shown in Fig. 2C., hemagglutination was totally abolished in FLL476 when compared to hemagglutination titers of 32 and 64 for 33277 and FLL476C’, respectively.

### VimF can Modulate the Invasive Capacity of *P. gingivalis*

HeLa cells incubated with *P. gingivalis* FLL95 and FLL476 showed a decrease in invasion of approximately 45% compared to the wild-type (Fig. 2D). *P. gingivalis* FLL95 complemented with the wild-type gene restored its invasive capacity similar to the parent strain (data not shown).

### The Cell Surface is Altered in the *vimF*-defective Isogenic Mutant

**Figure 3:**
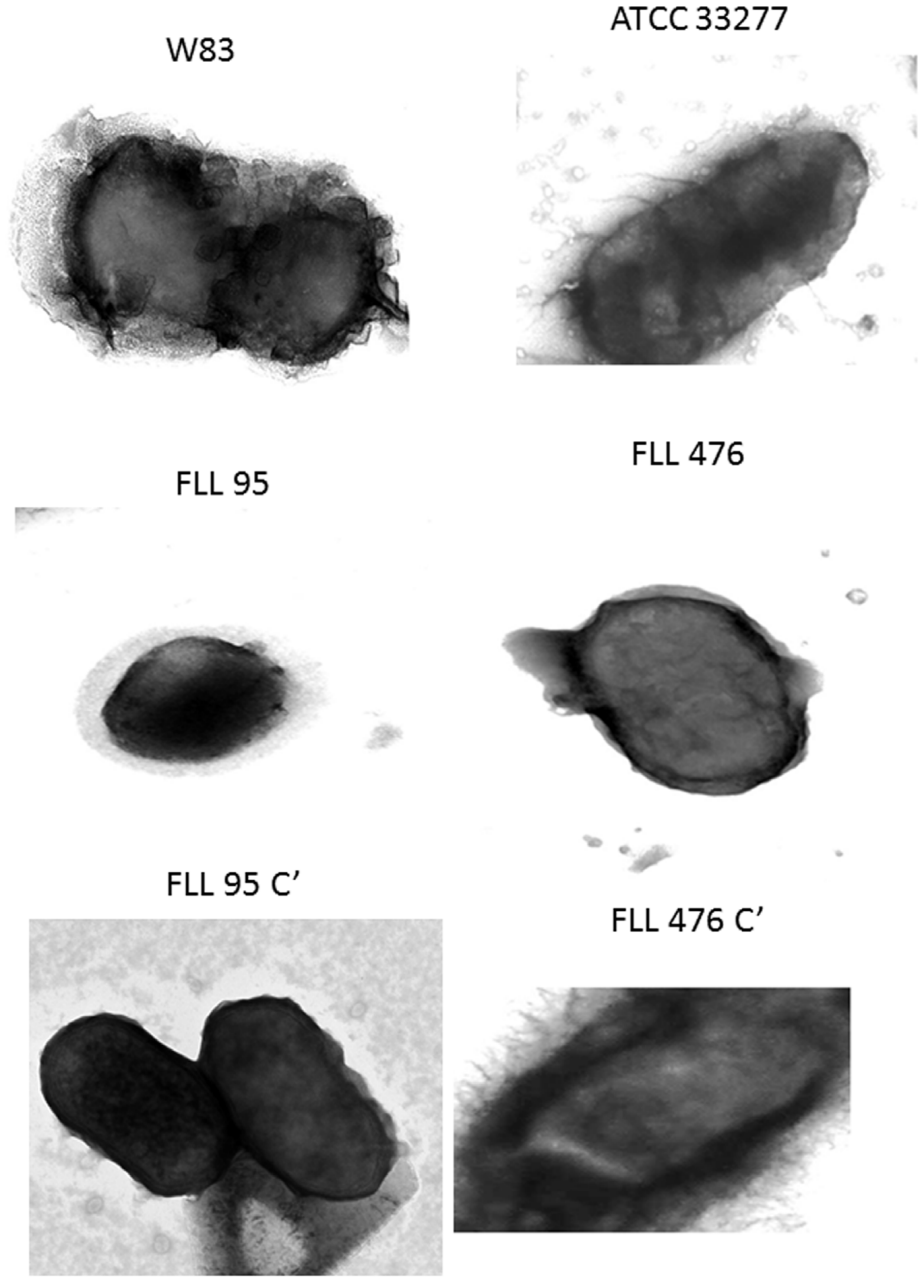
Electron micrograph showing changes in surface structures of *P. gingivalis* ATCC 33277 and W83. Bacterial cells grown to the log phase (OD_600_ of 0.7–0.9) were processed for electron microscopic examination using formvar-carbon coated grids (500 mesh) and were examined using Philips Tecnai 12 TEM. Fimbriae were lacking in the *vimF* mutant FLL476 when compared with the wild ATCC33277 and the complemented strain FLL476C’. A thick glycocalyx along with vesicles and a well-defined outer membrane was observed in W83. FLL95 showed hazy outer membrane with reduced visicles. In the complemented strain FLL95C’ the outer membrane morphology was restored.

Electron microscopy was used to evaluate the cell surface ultra-structure of the wild-type compared to the *vimF*-defective mutants. The wild-type W83 parent strain revealed well defined outer membrane with outer membrane vesicles (Fig. 3) that were missing in the isogenic mutant FLL95. The outer membrane and membrane vesicles was restored in the complemented strain, FLL95C’. Electron micrographs of ATCC 33277 and its isogenic mutant FLL476 revealed a modified cell surface that was devoid of fimbria in the FLL476 mutant. The wild-type phenotype was largely restored in the complemented strains.

### Cloning, Expression and Purification of rVimF

**Figure 4:**
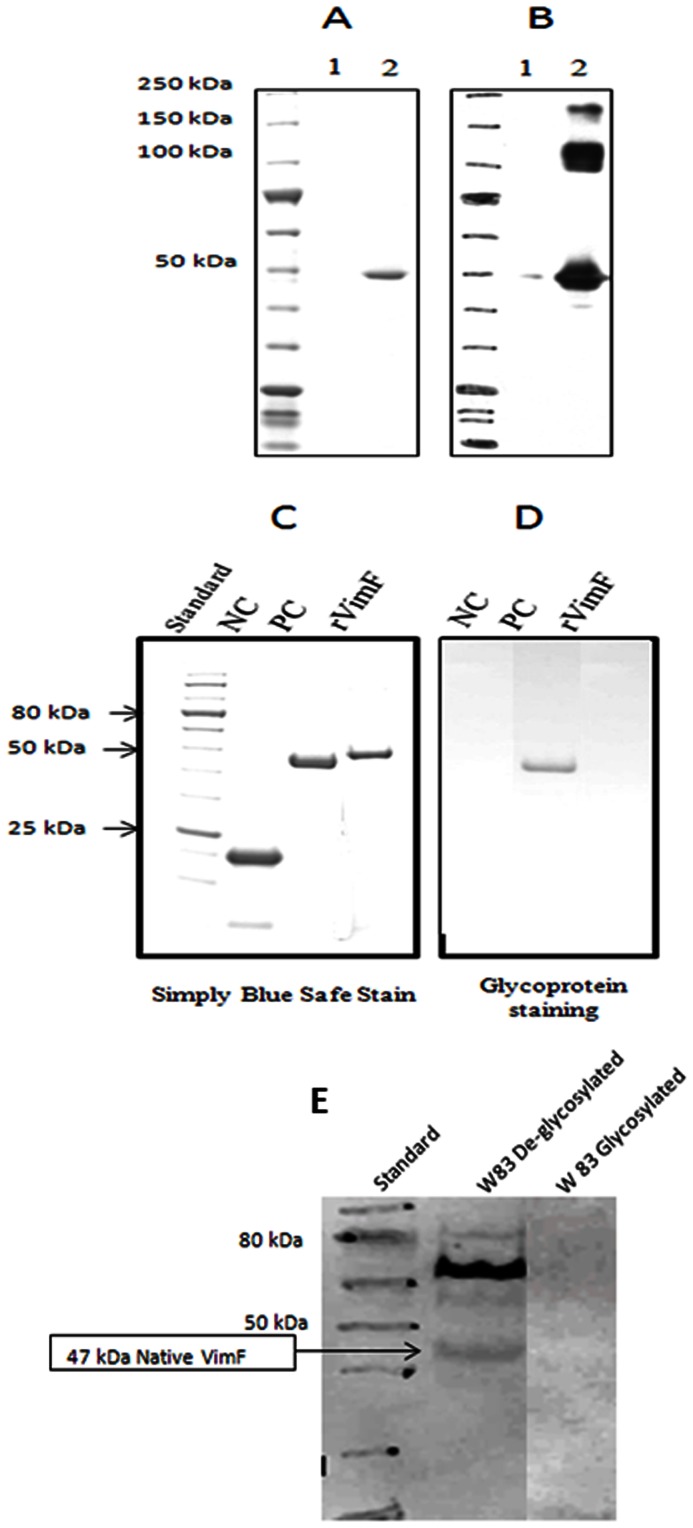
1D and 2D SDS-PAGE of rVimF. Purified rVimF was denatured in an LDS-containing buffer with DTT and heated for 10 min, and subjected to SDS-PAGE analysis. **A**. Simply Blue Safe stain of rVimF at 2 different concentrations: lane 1–0.4 µg and lane 2–1.2 µg. **B.** Western blot using anti-rVimF antibody against purified rVimF showed reacting bands at 50, 100 and 200 kDa. **C.** Simply blue safe stain of rVimF with horseradish peroxidase as positive (PC) and soybean trypsin inhibitor as negative (NC) controls for glycoproteins. **D.** Identical gel in panel C stained by periodic acid-Schiff (PAS) method for glycoproteins. **E.** Western blot using anti-rVimF showed a 47 kDa reactive band only when total proteins of W83 were deglycosylated and not with native (glycosylated) forms.

The *vimF* ORF was cloned into a His-tag containing *E. coli* expression vector. The expected 50 kDa rVimF (47 kDa VimF and 3 kDa for the 6X Histidine tag) was not observed to be secreted but was shown to co-purify with GroEL (60 kDa) only in cell lysates. The purified rVimF protein showed a single band near the 50 kDa region (Fig. 4A). However western blot using anti-rVimF antibody showed reactive bands also at 100 kDa and 200 kDa regions (Fig. 4B). These two bands corresponding to the multimeric forms of rVimF were confirmed using anti-rVimF antibodies and mass spectroscopy (data not shown). 2D gel electrophoresis of the purified rVimF showed isoforms_near the 50 and 100 kDa regions which were identified as VimF by mass spectroscopy (data not shown). Since glycosylation of proteins is a common cause for the isoforms observed in 2D gels, we used a glycoprotein stain to test whether rVimF was glycosylated. When compared with positive and negative controls for glycoprotein staining, rVimF did not take up the glycoprotein stain (Fig. 4D and 4C).

### The Native VimF may be Post-translationally Modified

Polyclonal antibodies raised against the gel purified rVimF immunoreacted with multiple proteins bands representing the rVimF multimeric forms (Fig. 4B). Immune serum did not immunoreact with any other *E. coli* protein (Fig. 4B). Similar immunoreactive bands observed with the immune serum were missing using the pre-immune serum in Western blot analysis (data not shown). To determine if the antibodies against the recombinant VimF protein can recognize the native protein, cell lysates from *P. gingivalis* were separated by SDS-PAGE and probed with the anti-rVimF antibodies. As shown in Fig. 4E, immunoreactive bands from the *P. gingivalis* cell lysates were missing using the immune serum. However, immunoreactive bands with sizes of 47, 60 and 80 kDa were observed when cell lysate proteins from W83 were first deglycosylated then separated by SDS-PAGE and probed with the anti-rVimF antibodies (Fig. 4E). The 47 kDa band corresponds to native VimF. Taken together, these results suggest that differences may exist between the glycosylation of the native and recombinant VimF protein. It is noteworthy that the rVimF was negative for glycoprotein stain (Fig. 4D).

### rVimF shows Galactosyltransferase Activity

**Figure 5:**
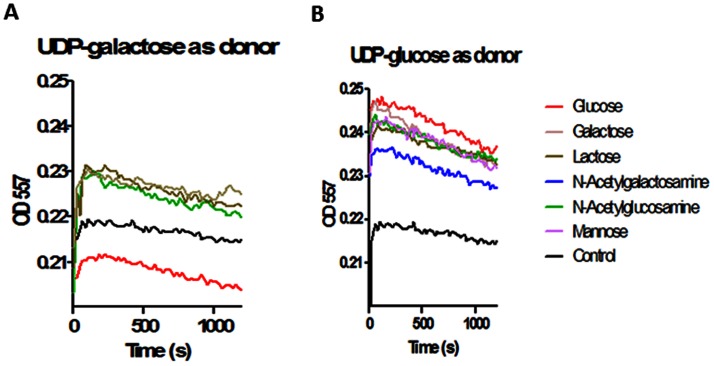
Screening for donor and acceptor substrates. Change of absorbance at 557 nm with time for **A**. UDP-galactose as donor and **B**. UDP- glucose as donor was plotted using various sugars as acceptors. Lysates (100 µl) of *E. coli* expressing rVimF was used as enzyme source and, lysates of *E. coli* Top 10 cells was used for negative control. The reaction mix contained 2 mM phosphate, pH 8, 0.01 mM phenol red, 0.1 mM MnCl_2_, and 10 mM acceptor sugars. A lower OD_557_ value was observed when UDP-galactose was used as the donor.

VimF is annotated as a putative glycosyltransferase type 1 (http://oralgen.lanl.gov). Thus the activity of rVimF was evaluated using a calorimetric assay [25] that exploits the lowering of pH resulting from the release of protons associated with glycosyltransferase activity. The change in pH is detected spectrophotometrically using a phenol red indicator. A calibration curve using known concentrations of HCl was used to establish the relationship between proton release and decrease in OD_557_ (data not shown). Commercially available UDP-galactose or UDP-glucose as donor substrate and, glucose, galactose, mannose, *N*-acetylglucosamine or *N*-acetylgalactosamine as acceptor substrate, was used to screen for rVimF glycosyltransferase activity. As shown in Fig. 5, the largest initial drop in OD_557_ was observed when UDP-galactose (Fig. 5A) was used as the donor substrate as compared to UDP-glucose as donor (Fig. 5B). Among the acceptor sugars used for UDP-galactose as donor, glucose followed by *N-*acetyglucosamine showed the lowest OD_557_ in the time course experiments suggesting their acceptor function in the presence of rVimF to these two sugars. Therefore for activity assays we chose to use *N*-acetylglucosamine as it is also the commonest acceptor used for commercially available β-1,4-galactosyltransferases (Sigma, St. Louis, MO) which we chose as positive control. Lysate from *E. coli* Top 10 cells was used as negative control.

**Figure 6:**
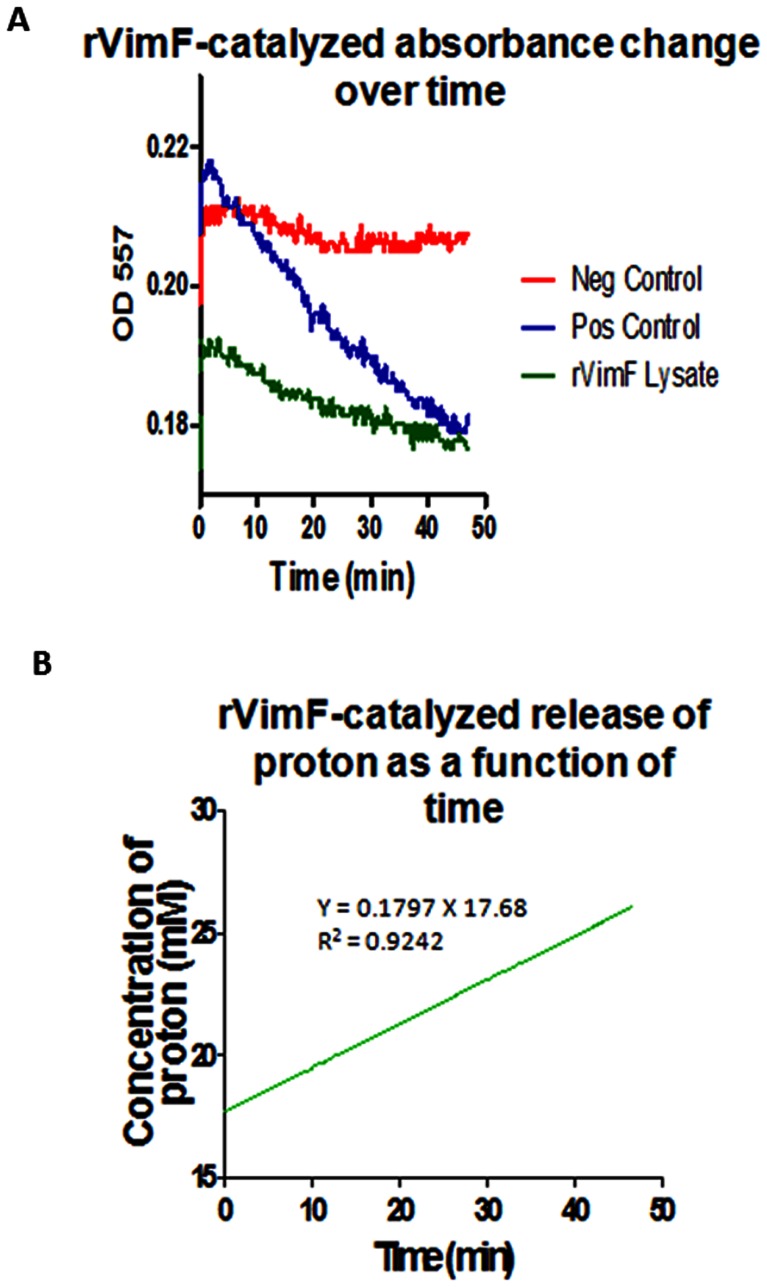
Galactosyltransferase activity of rVimF. Activity of rVimF compared with positive control (commercially available β- 1,4-galactosyltransferase) and Negative control (non-specific *E. coli* Top 10 lysate). 100 µl of whole cell lysates of *E. coli* containing pFLL477 expressing rVimF was added to reaction mix containing 2 mM phosphate, pH 8, 0.01 mM phenol red, 0.1 mM MnCl_2_, 10 mM *N*-acetylglucosamine and UDP-galactose was added to start the reaction. OD_557_ was measured for 50 minutes. **A**. Time course showing average of 3 independent assays. **B**. Activity of rVimF as a galactosyltransferase was calculated by converting change in OD_557_ to amount of proton release over time using the calibration curve in GraphPad Prizm 5 software.

Using UDP-galactose as donor and *N*-acetylglucosamine as acceptor the enzyme activity of rVimF was calculated and compared with a commercially available β-1, 4-galactosyltransferase. Fig. 6A shows a typical time trajectory of absorbance change corresponding to rVimF-catalyzed proton release (higher the proton release lower the OD_557_) in comparison to the positive and negative controls. Using the calibration curve, rVimF-catalyzed proton concentration change corresponding to the absorbance change was calculated and plotted as a function of time (Fig. 6B). A linear regression was performed (*R^2^* = 0.9242) and the slope was estimated to be 0. 1797. The enzyme activity of rVimF was calculated using the formula
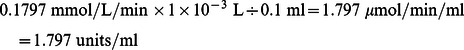



Enzyme activity was defined as the amount of enzyme needed to produce 1 µmol of proton per minute. Using the same formula, enzyme activities of the positive and negative control was calculated to be 5.0 U/ml and 0.6 U/ml, respectively. Activity of positive control, commercially available β-1,4-Galactosyltransferase, estimated by our assay corresponds well with prescribed activity of ≥0.6 U/ml suggested by the manufacturer (Sigma, St. Louis, MO).

### FLL95 Gingipain Proenzyme did not Show Glycan Attachment

**Figure 7:**
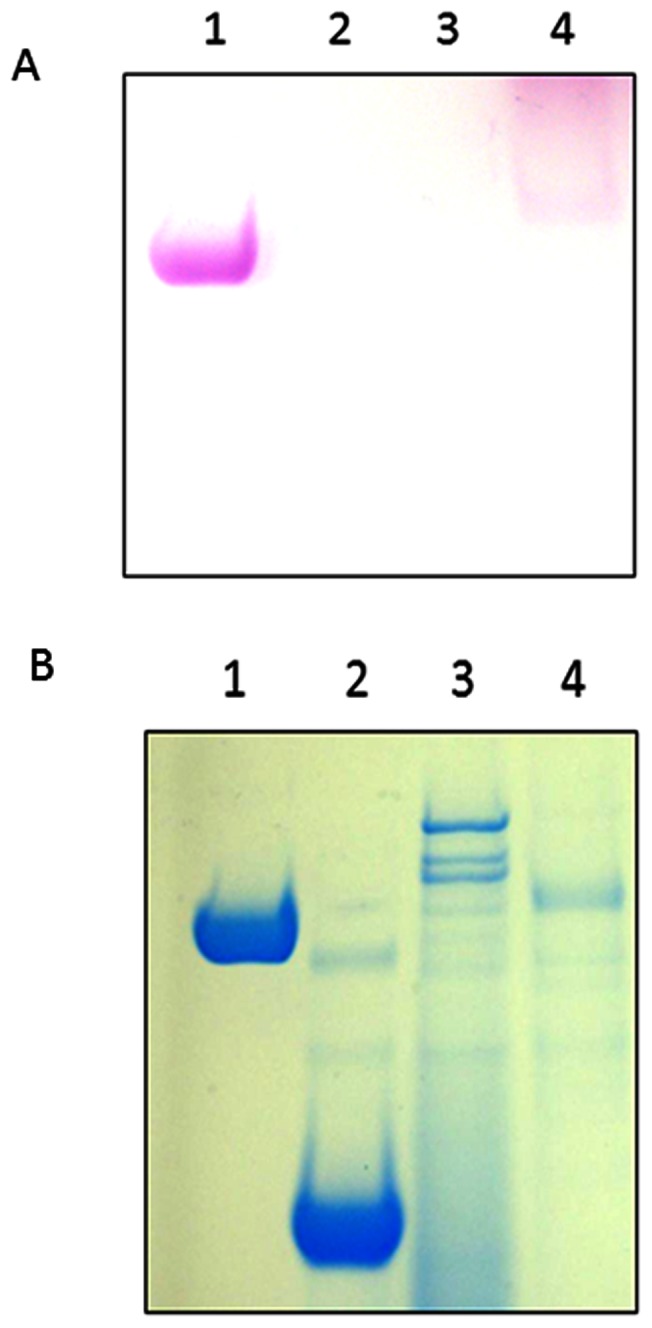
FLL95 Gingipain proenzyme is devoid of carbohydrate attachment. (**A**) Carbohydrate stain compared to (**B**) Simply Blue Stain of W83 catalytic domain and FLL95 proenzyme: lane 1, positive control using horseradish peroxidase provided by kit; lane 2, negative control using soybean trypsin inhibitor provided by kit; lane 3, FLL95 proenzyme; lane 4, W83 catalytic domain.

To further clarify the role of VimF in the glycosylation of gingipains, the presence of carbohydrates on the proenzyme gingipain species from FLL95 was determined using SDS-PAGE glycoprotein stain. As shown in Fig. 7A, no detectable band was observed in FLL95 in contrast to the gingipains from wild-type W83. For comparison a similar gel stained with SimplyBlue SafeStain (Fig. 7B) is shown. A more sensitive technique using methanolysis and silylation followed by GC-MS analysis of the TMS-methyl glycosides confirmed the absence of any detectable sugar moiety attached to the gingipain proenzyme species (data not shown). Taken together, these results suggest that the proenzyme species from the *vimF* defective mutant is devoid of any detectable glycan modification.

### rVimF-dependent Gingipain Glycosylation

**Figure 8:**
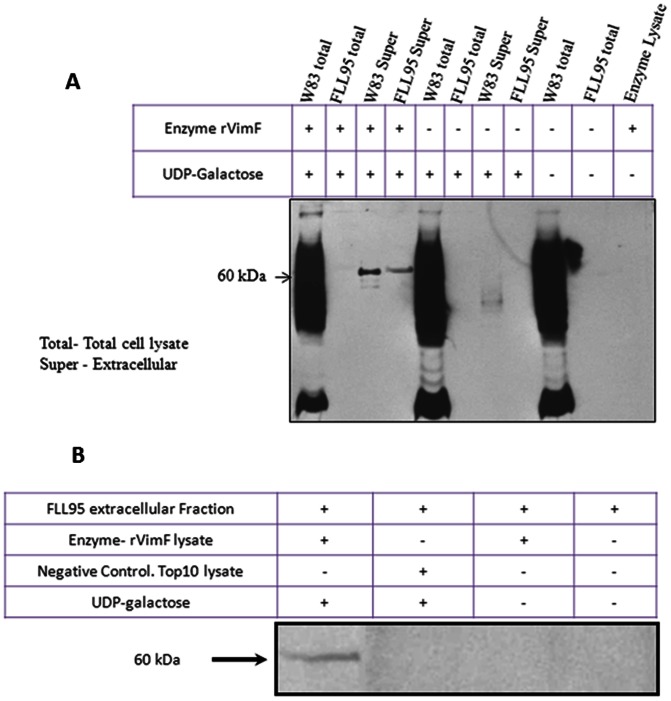
In-vitro galactosyltransferase assay. Total cell lysate or extracellular fractions from W83 and FLL95 were used as acceptor substrates, *E. coli* lysate carrying pFLL477 served as its enzyme source and UDP-galactose served as donor substrate. Western blots were probed with glycan specific mAb IB5. **A.** 60 kDa band appeared when both UDP-galactose and rVimF enzyme lysate were present. **B**. Using extracellular fractions of FLL95 as acceptor substrate a 60 kDa band was seen only when rVimF lysate and UDP-galactose were present. Negative control using Top 10 *E. coli* lysate did not show the 60 kDa band.

In the *P. gingivalis vimF*-defective (FLL95) mutant the presence of the gingipain proenzyme species and their lack of immunoreactivity to mAb 1B5 suggest a glycosylation defect [12]. With the preference of rVimF to transfer galactose to *N*-acetylglucosamine, we evaluated its ability to transfer galactose to acceptor proteins in *P. gingivalis*. In the presence of rVimF and UDP-galactose, a 60 kDa band that immunoreacted with the glycan specific mAb 1B5 was observed in the extracellular fraction of FLL95 (Fig. 8A). This band was missing in the absence of UDP-galactose or when lysates of *E.coli* TOP 10 cells were used instead of rVimF in the reaction mixture (Fig. 8B). Using mass spectroscopy this 60 kDa band was identified as Rgp progingipain. A similar 60 kDa band in addition to a 45 kDa band was observed in the extracellular fraction of W83 only when rVimF was present in the reaction mixture.

## Discussion

Proteolytic processing and glycosylation are important components in the maturation/activation/anchorage of the gingipains in *P. gingivalis* [38]. In this report we have used both genetic and biochemical approaches to further confirm the role of specific bacterial host factors in gingipain biogenesis. Several recent studies have identified the involvement of many nongingipain genes in this process [39]–[44]. In addition a conserved C-terminal domain (CTD) is essential for the secretion and attachment of the gingipains to the cell surface [45]. Collectively, an emerging picture from these studies suggests a complex process that is facilitated by the appropriate glycosylation of the gingipains.

Glycosylation is a recently identified post-translational modification of proteins in prokaryotes [19]. This process which involves the enzymatic attachment of a glycan moiety to a protein is known to influence biological properties like activity, solubility, folding, conformation, stability, half-life, and/or immunogenicity [46], [47]. Since the initial report of the S-layer glycoprotein of archaea *Halobacterium halobium* (salinarum), some of the well-known examples of bacterial glycoproteins belong to genera *Campylobacter*, *Mycobacterium*, *Neisseriae*, *Pseudomonas*, *Chlamydiae*, *Escherichia* and *Porphyromonas* [7], [19]. In *P. gingivalis,* a defect in several GTases including Rfa, UgdA, GtfA, GtfB have been shown to affect polysaccharide biosynthesis that have resulted in decreased gingipain activities [11], [17], [18]. VimF, which is annotated as a putative glycosyltransferase (www.oralgen.lanl.gov) has been shown to be involved in maturation of gingipains, hemolysis, hemagglutination and fibronectin cleavage similar to some of the other GTase-defective mutants previously described [12], [48]. In this study, several of these phenotypic characteristics were confirmed in a different *P. gingivalis* genetic background. These observations further support the vital role of VimF in the pathogenesis of *P. gingivalis*.

Compared to the wild-type, the growth rate and gingipain activity were reduced in the *vimF*-defective mutants in both genetic backgrounds. However, the gingipain activity in *P. gingivalis* FLL476 was reduced by approximately 60% compared to more than 90% reported in *P. gingivalis* FLL95 [12]. This observation could suggest the role of additional factor(s) that may be involved in the maturation/glycosylation of the gingipains. For example, a functional homologue of *vimF* or an alternate glycosyltransferase, which is more effective in the *P. gingivalis* 33277 genetic background could be responsible for the increased activity. There are multiple glycosyltransferases reported in *P. gingivalis* (http://www.cazy.org/), however the substrate specificity for several of these enzymes is unknown.

The invasive capacity of the *vimF*-defective isogenic mutant in multiple genetic backgrounds was reduced compared to the wild-type. This was not unexpected, given the cell surface alterations in the *vimF*-defective mutants. This alteration in cell surface is also thought to contribute for the increased biofilm formation observed in FLL476. Similar observations have been reported [16], [49]. The role of fimbria in host cell invasion by *P. gingivalis* is well documented [50]. In the *vimF*-defective isogenic mutant, the phenotypic expression of fimbria appeared to be altered. In addition it is known that a defect in LPS biosynthesis in P. gingivalis can influence attachment of the gingipains to the cell surface, autoaggregation, and biofilm formation. These phenotypic properties are known to be associated with P. gingivalis invasive capacity.

We have observed that the His-tagged rVimF, a 50 kDa protein, is homotetrameric and, on 2D gel showed isoforms. Purified rVimF was observed as a single band (Fig. 4A). However, the same gel, when subjected to western blot analysis and probed with anti-rVimF antibody showed immune reactivity to three different bands corresponding to 50 kDa, 100 kDa and 200 kDa (Fig. 4B). This seemingly conflicting observation is possible as rVimF may not be completely denatured in the presence of 10% SDS. The multimeric rVimF forms are consistent with the 200 kDa band observed in a native gel. Isoforms observed on the 2D gel is likely due to post-translational modifications of the rVimF protein. The type of modification is under further investigation.

In *P. gingivalis,* the anti-rVimF antibody immunoreacted with multiple protein bands only after deglycosylation. The 47 kDa protein band is the expected size of the VimF product and likely suggests that it’s a glycoprotein. The 60 and 80 kDa immunoreactive bands may have conserved domains that can cross react with the anti-rVimF antibody although we cannot rule out mutimeric forms of VimF. Further investigation of their identity and possible function is underway.

The *P. gingivalis vimF* gene encodes for a 47 kDa protein that has galactosyltransferase activity. This, possible multimeric protein, was demonstrated to have the ability to transfer UDP-galactose to *N*-acetylglucosamine. VimF, which may also be a glycoprotein, is suggested by these studies to play a specific role in gingipain glycosylation. In contrast to the *vimA*-defective mutant which only had the RgpB gingipain cell associated, or other GTase-defective mutants that are missing any cell-associated gingipain, the *vimF*-defective mutants had both cell and extracellular associated inactive forms of the gingipains [43], [44], [51]. Throughout all the growth phases, no activation of the gingipains was observed. Variation in the glycosylation profile of the gingipains including the missing phosphorylated branched mannan was also noted [44], [52] in the *vimF* mutants. In the presence of rVimF and UDP-galactose, a 60 kDa band identified as RgpB regained its missing phosphorylated branched mannan. This could imply that galactose is important for the addition of the glycan moiety carrying the phosphorylated branched mannan. The monosaccharide composition of the gingipains from *P. gingivalis* W50 is known to include arabinose, rhamnose, fucose, mannose, galactose, glucose, GalNAc, GlcNAc, and N-acetylneuraminic acid [13]. There also appears to be common steps in the synthesis of LPS and APS and the maturation of the gingipains [13], [52], [53]. A bioinformatic analysis of the Rgp-gingipains predicted two potential O-linked and 15 potential N-linked glycosylation sites for RgpA. This is in contrast to RgpB that was predicted to have 6 N-linked and no O-linked glycosylation sites. Reported elsewhere, the sugars in RgpA are thought to be present predominately in O-linked chains with the monosaccharide GalN(Ac) linked to Ser/Thr [13]. Most N-linked glycan chains occur via *N-*acetylglucosamine attached to asparagine and followed sequentially by hexoses such as galactose [54]. The results from this study suggest that galactose is vital for the growing glycan chain. Because the gingipains from the *vimF*-defective mutant were missing any detectable carbohydrate modifications, this raises questions regarding the monosaccharide protein link. While we cannot rule out an *N-*acetylglucosamine link, as observed in *Haemophilus,* it is likely that galactose can occupy these N-linked sites [55]. It is also unclear if the attachment of these glycans occurs either sequentially or en-block. Further, in these experiments it was unclear if the gingipain could complete the maturation process and gain proteolytic activity. This is under further investigation in the laboratory.

Our observation that the inactive proenzyme species can be cell associated in the *vimF*-defective *P. gingivalis* strain raises questions on the specific glycosylation requirement for attachment. Further, based on previous reports, the posttranslational proteolytic processing of the gingipains involves multiple enzymes for their activation. RgpA and Kgp are known to require a surface located carboxypeptidase for activation in contrast to RgpB which is known to require a novel C-terminal signal peptidase [56], [57]. It is clear from our studies that the appropriate glycosylation may be a prerequisite for proteolytic processing and the addition of galactose may occur early in the sequence.

In conclusion, we have presented *in vitro* evidence for posttranslational regulation of proteolytic activity in *P. gingivalis*. *In vitro* glycosyltransferase activity for rVimF was observed using UDP-galactose and *N*-acetylglucosamine as donor and acceptor substrates, respectively. Further, in the presence of the rVimF protein and UDP-galactose, the glycosylation of the RgpB proenzyme was restored. Together, these observations suggest the VimF glycoprotein is a galactosyltransferase that may be specific for gingipain glycosylation. Moreover, galatose is vital for the growing glycan chain. This model system will facilitate a more careful evaluation of glycosylation occurring in gingipain biogenesis in *P. gingivalis.* Components of this system could possibly be an important therapeutic target.
