# Correction: In *Porphyromonas gingivalis* VimF Is Involved in Gingipain Maturation through the Transfer of Galactose

**DOI:** 10.1371/journal.pone.0223145

**Published:** 2019-09-23

**Authors:** Arun S. Muthiah, Wilson Aruni, Antonette G. Robles, Yuetan Dou, Francis Roy, Hansel M. Fletcher

Following publication of this article [1], concerns were raised regarding splicing of blot images in Figs 4 and 8. The authors provide clarifications regarding the assembly of the figures as follows:

- In Fig 4C, lanes three and four are spliced together because two lanes were removed from the original image.
- In Fig 4D, lanes two and three are spliced together because two lanes were removed from the original image.
- In Fig 4E, lanes two and three are spliced together because two lanes were removed from the original blot image.
- In Fig 8B, lanes one and two are taken from one blot, lanes three and four are taken from a separate blot run in a repeat experiment using a different exposure; the two blot images are spliced together. Furthermore, lanes three and four are spliced together because four lanes were removed from the original blot image.

The authors acknowledge that in Figs 4D and 8B there is the appearance of a vertical discontinuity between lanes 1 and 2 although these samples were run in adjacent lanes on the original gels. The results are supported by the underlying blots, which are provided as Supporting Information.

A revised Fig 4 is provided in which splicing together of separate lanes from the same blot is marked with a black line. Please see the revised Fig 4 and updated figure legend here.

**Fig 4:**
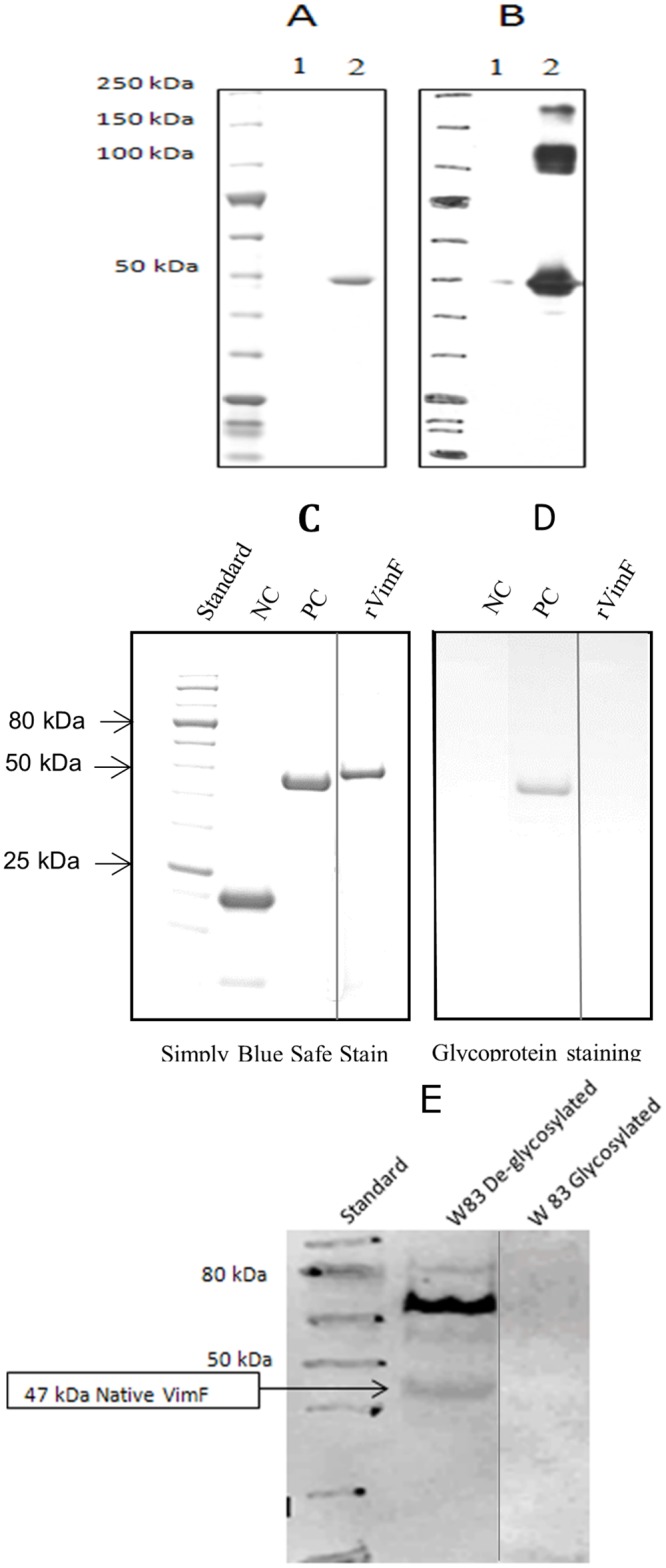
1D and 2D SDS-PAGE of rVimF. Purified rVimF was denatured in an LDS-containing buffer with DTT and heated for 10 min, and subjected to SDS-PAGE analysis. **A.** Simply Blue Safe stain of rVimF at 2 different concentrations: lane 1–0.4 μg and lane 2–1.2 μg. **B.** Western blot using anti-rVimF antibody against purified rVimF showed reacting bands at 50, 100 and 200 kDa. **C.** Simply blue safe stain of rVimF (lane 4 was spliced with rest of the lanes) with horseradish peroxidase as positive (PC) and soybean trypsin inhibitor as negative (NC) controls for glycoproteins. Lane 4 was spliced from the same gel image. **D.** Identical gel in panel C stained by periodic acid-Schiff (PAS) method for glycoproteins (rVimF in lane 3 was spliced to NC and PC). Lane 3 was spliced from same gel image. **E.** Western blot using anti-rVimF showed a 47 kDa reactive band only when total proteins of W83 were deglycosylated and not with native (glycosylated) forms. Lane 3 was spliced from same gel image.

A revised Fig 8 is provided in which lanes from different blots are shown in separate panels and splicing together of separate lanes from the same blot is marked with a black line. Please see the revised Fig 8 and updated figure legend here.

**Fig 8:**
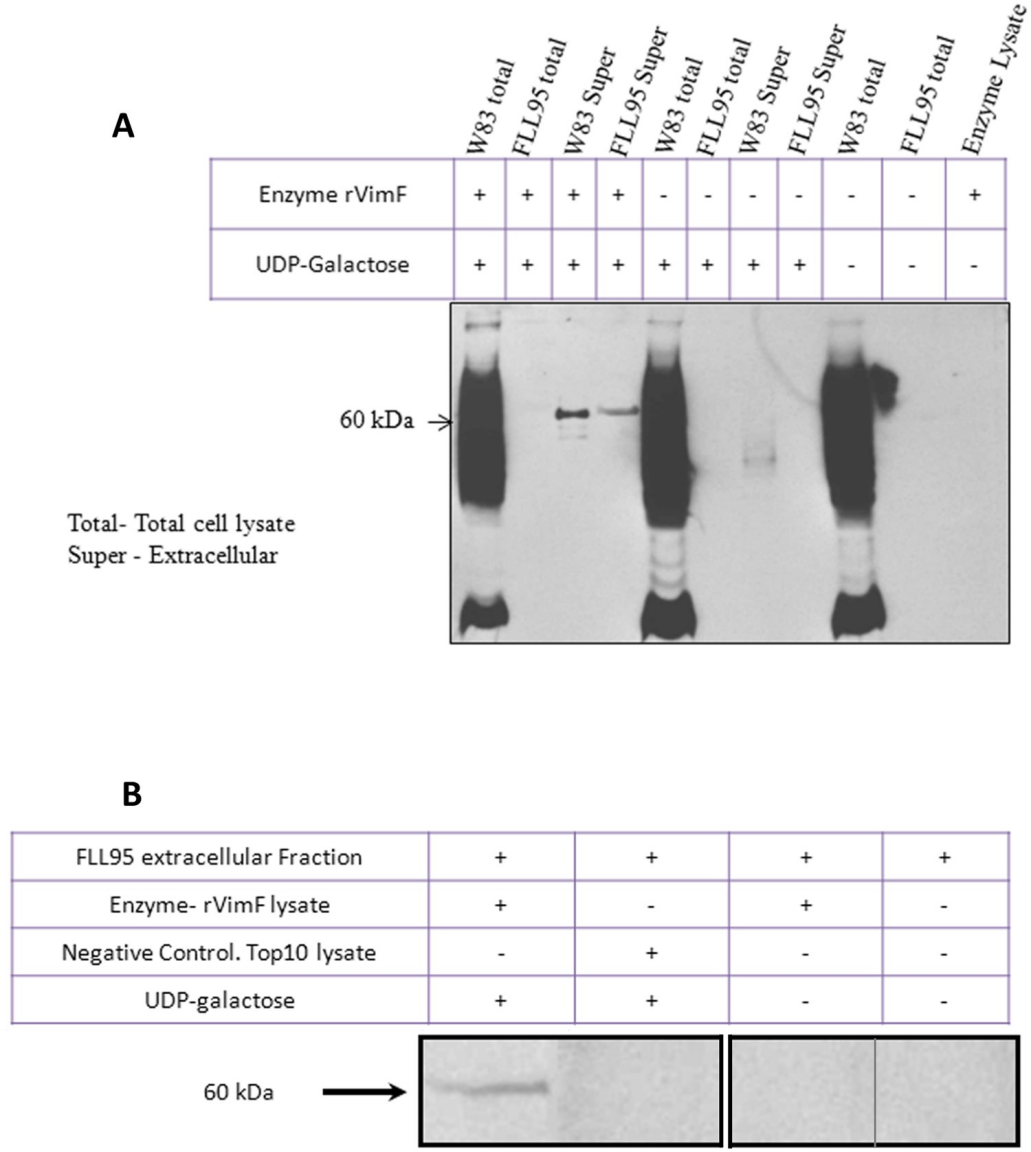
In-vitro galactosyltransferase assay. Total cell lysate or extracellular fractions from W83 and FLL95 were used as acceptor substrates, *E*. *coli* lysate carrying pFLL477 served as its enzyme source and UDP-galactose served as donor substrate. Western blots were probed with glycan specific mAb IB5. **A.** 60 kDa band appeared when both UDP-galactose and rVimF enzyme lysate were present. **B.** Using extracellular fractions of FLL95 as acceptor substrate a 60 kDa band was seen only when rVimF lysate and UDP-galactose were present. Negative control using Top 10 *E*. *coli* lysate did not show the 60 kDa band. Lanes 1 and 2 are from a single gel. Lanes 3 and 4 are from a different gel using a different exposure. Lanes 3 and 4 were spliced together from the same gel image.

The underlying blots for Figs 4 and 8 are provided as Supporting Information (S1 File).

## Supporting information

S1 File Underlying blots for Figs 4 and 8. (ZIP)
