# Supplementary material for: Correction: In Porphyromonas gingivalis VimF Is Involved in Gingipain Maturation through the Transfer of Galactose
Source: PLoS One. 2019 Sep 23;14(9):e0223145. doi: 10.1371/journal.pone.0223145 (PMC6756511; doi:10.1371/journal.pone.0223145)
Supplement: S1 File — (ZIP) [file pone.0223145.s001.zip › Original Figures 4 and 8.pdf]

Slide1: Original figure 4A and 4B

Figure 4A, SDS-PAGE analyses of rVimF purification (protein gel, simple blue staining). Lane1: Protein marker; Lane 2, Washing buffer eluate 2 µL; Lane 3, Washing buffer 6 µL; Lane 4, Elution buffer 2 µL; Lane 5, Elution buffer 6 µL.

Figure 4B, Western-blot of protein gel (Fig 4A) by using anti-rVimF antibody (Scan picture of the western-blot film). All the lanes' samples are as same as Fig 4A.

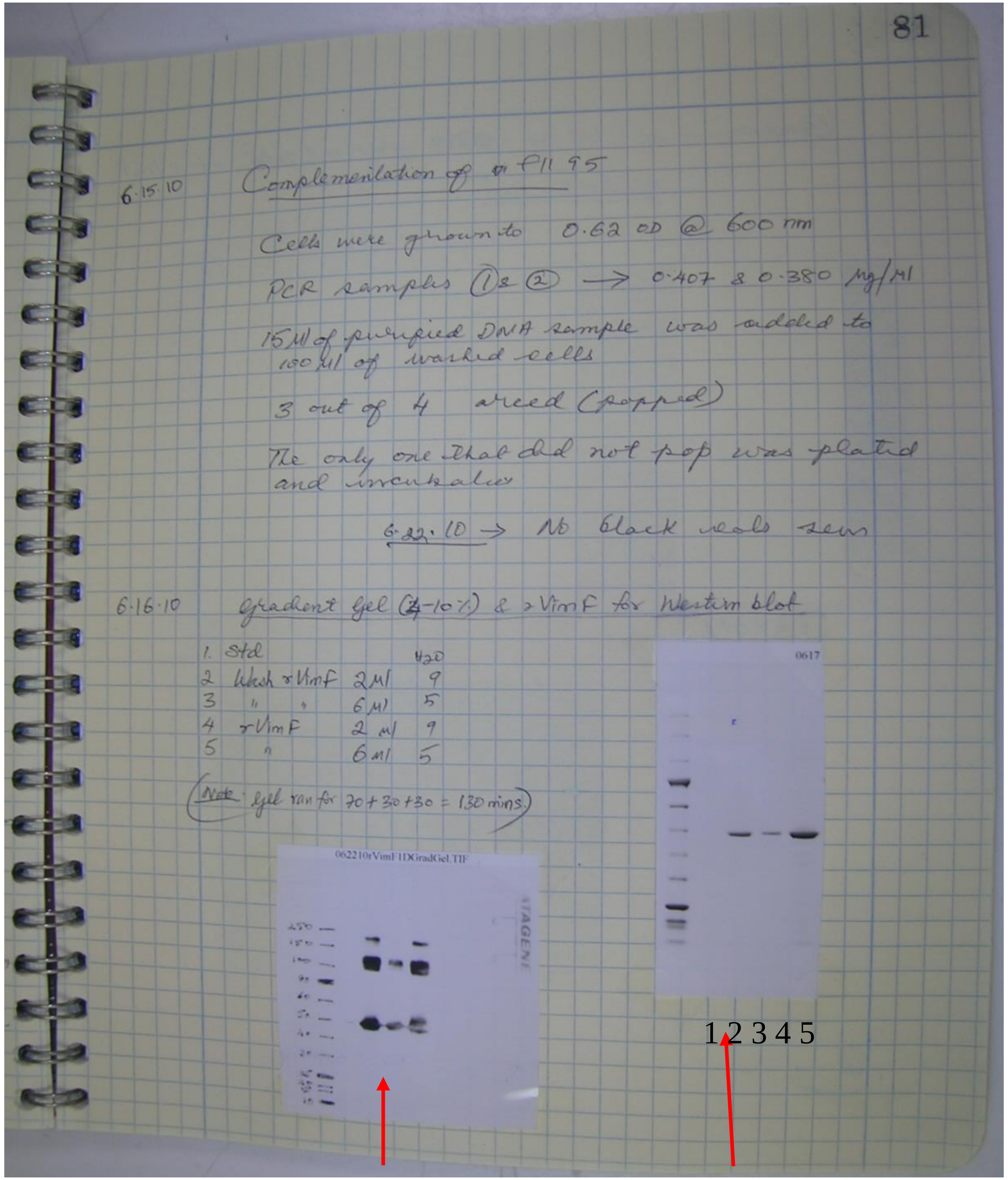

Original Fig. 4B Original Fig. 4A

Slide2: Original figure 4C

Figure 4C, Simply blue safe stain of rVimF with horseradish peroxidase as positive (PC) and soybean trypsin inhibitor as negative (NC) controls for glycoproteins.(protein gel, simple blue staining). Lane1: Protein marker; Lane 2, Negative control; Lane 3, Positive control; Lane 4, Complete reactions; Lane 5, Reaction without enzyme; Lane 6, purified rVimF; Lanes 7~10, Samples from previous reactions. The lanes shown in Figure 4C were marked by arrows.

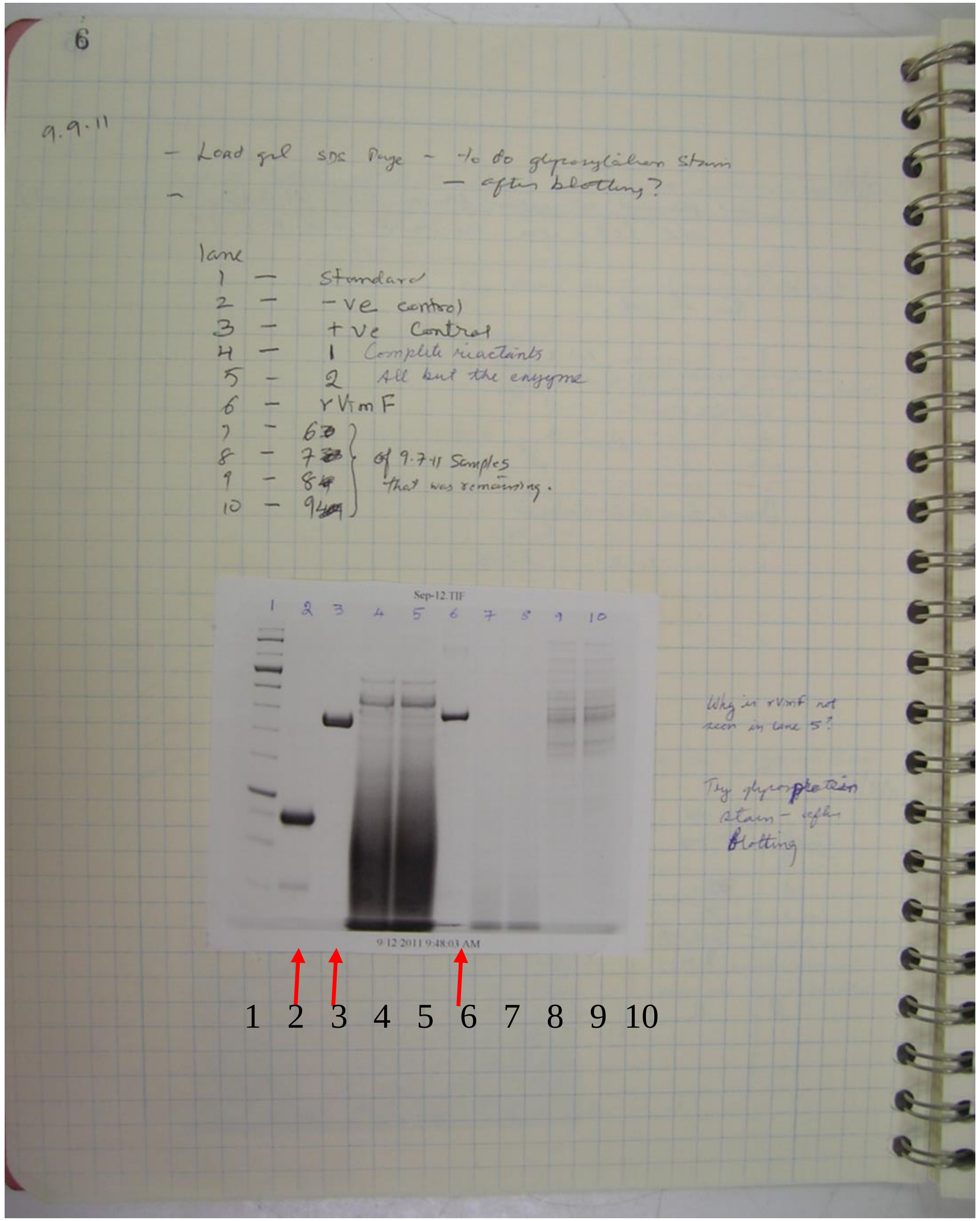

Original figure 4C

Slide3: Original figure 8A

Figure 8A , Western-blot of *in vitro* galactosyltransferase assay by using glycan specific mAb IB5 antibody. Lane 1, protein marker; Lane 2, W83 lysate control; Lane 3, FLL95 lysate; Lane 4, W83 supernatant; Lane 5, FLL95 supernatant; Lane 6, W83 lysate control; Lane 7, FLL95 lysate; Lane 8, W83 supernatant; Lane 9, FLL95 supernatant; Lane 10, W83 lysate; Lane 11, FLL95 lysate; Lane 12, Enzyme lysate. Lanes 2~5, and lane 12 containing rVimF.

Pic 1, Original western-blot film (Fig 8A); Pic 2, Original membrane containing all samples for western-blot

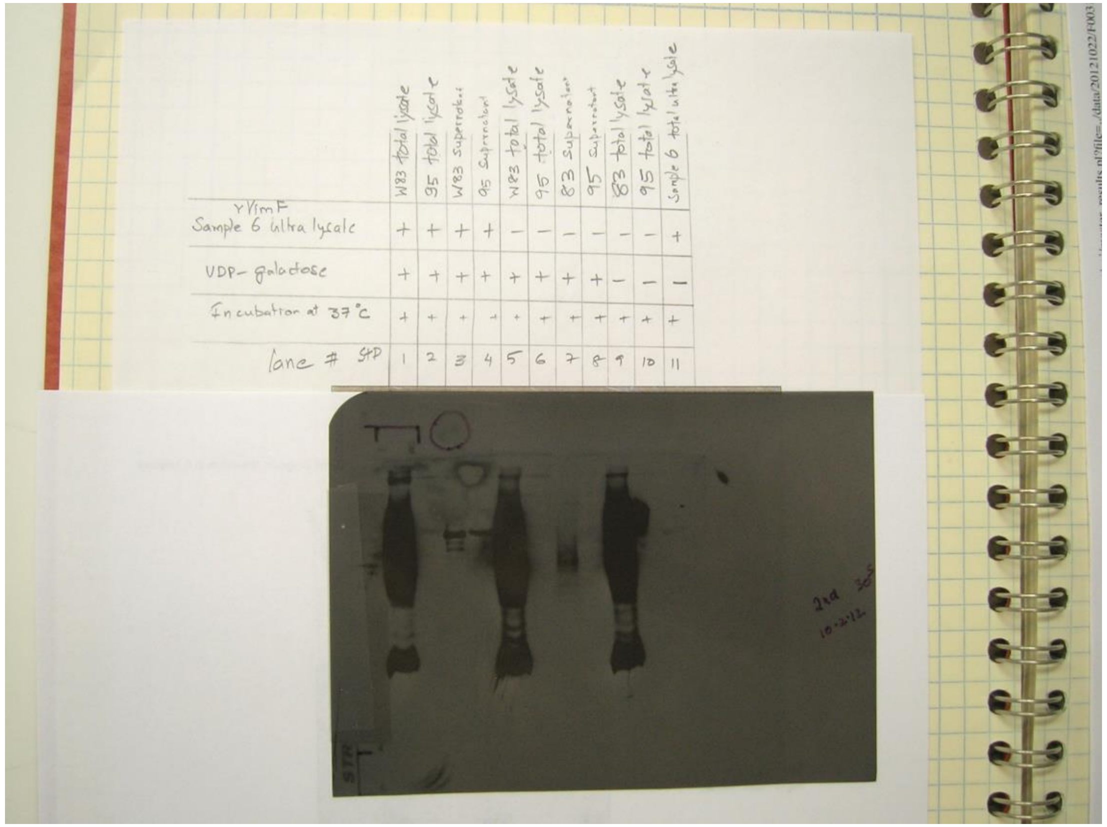

1

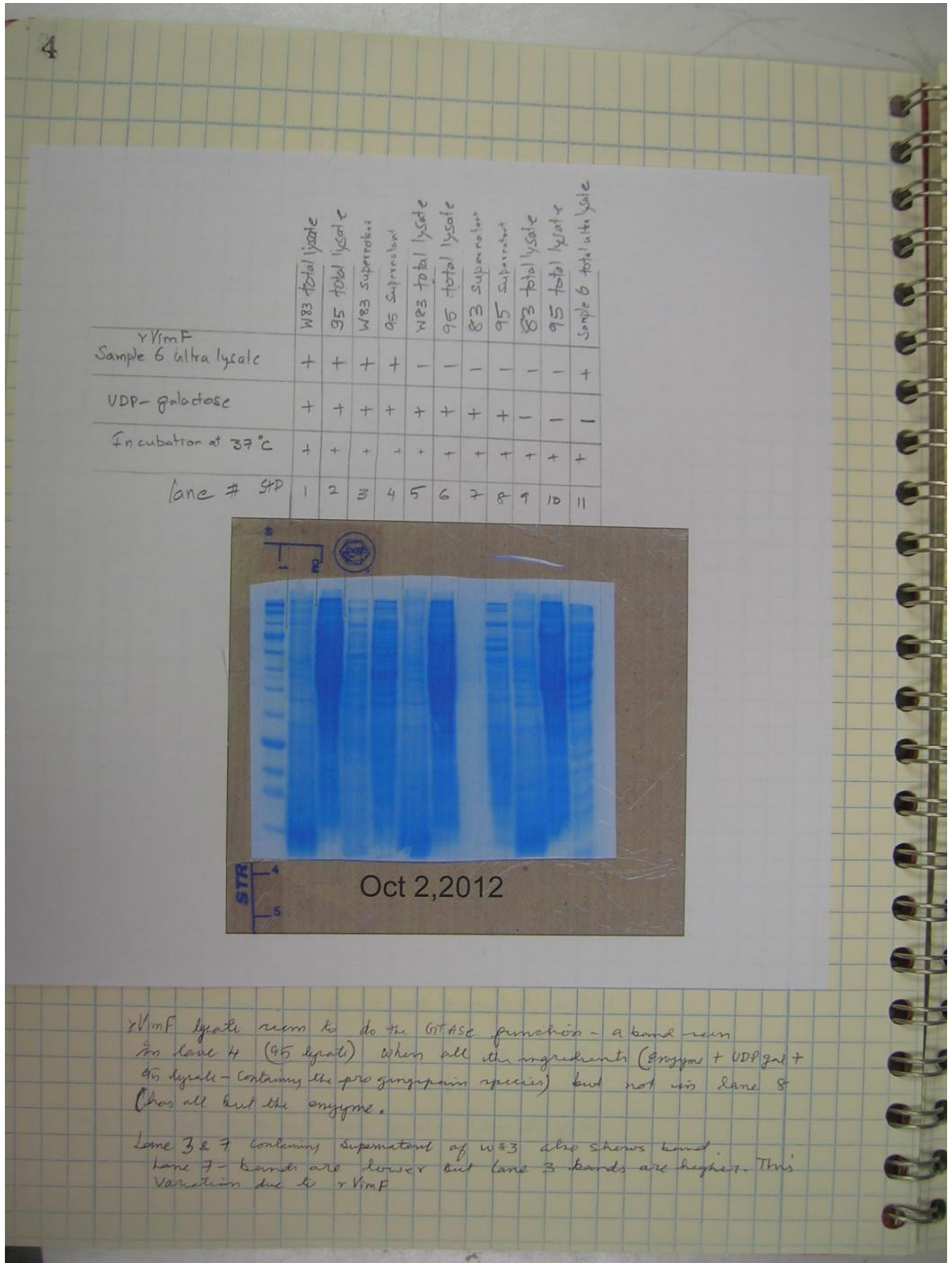

2
