# Supplementary material for: Correction: In Porphyromonas gingivalis VimF Is Involved in Gingipain Maturation through the Transfer of Galactose
Source: PLoS One. 2019 Sep 23;14(9):e0223145. doi: 10.1371/journal.pone.0223145 (PMC6756511; doi:10.1371/journal.pone.0223145)
Supplement: S1 File — (ZIP) [file pone.0223145.s001.zip › Uncropped Images for Fig 4 and 8.pptx]

## Slide 1
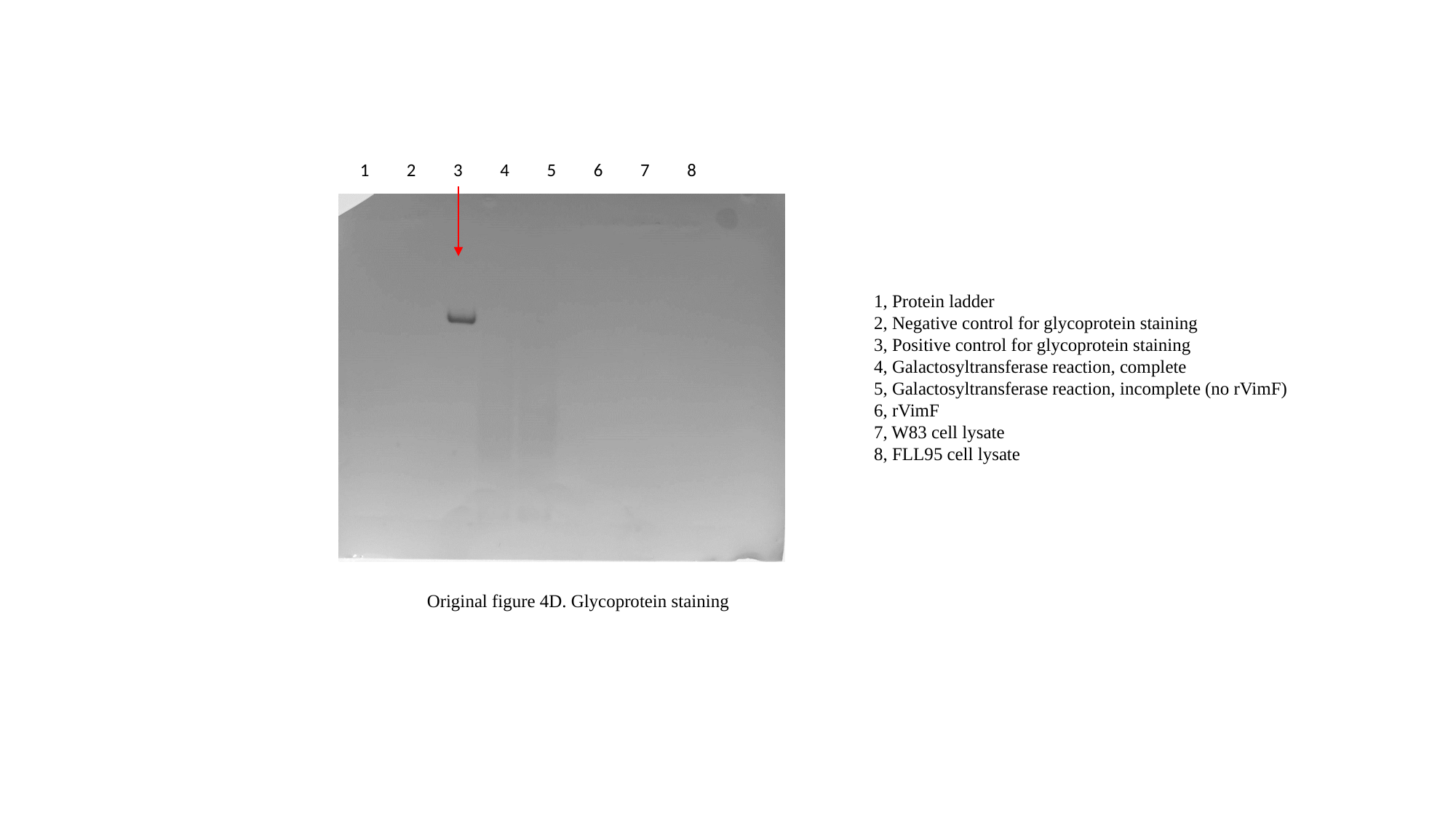

1 2 3 4 5 6 7 8
1, Protein ladder
2, Negative control for glycoprotein staining
3, Positive control for glycoprotein staining
4, Galactosyltransferase reaction, complete
5, Galactosyltransferase reaction, incomplete (no rVimF)
6, rVimF
7, W83 cell lysate
8, FLL95 cell lysate
Original figure 4D. Glycoprotein staining

## Slide 2
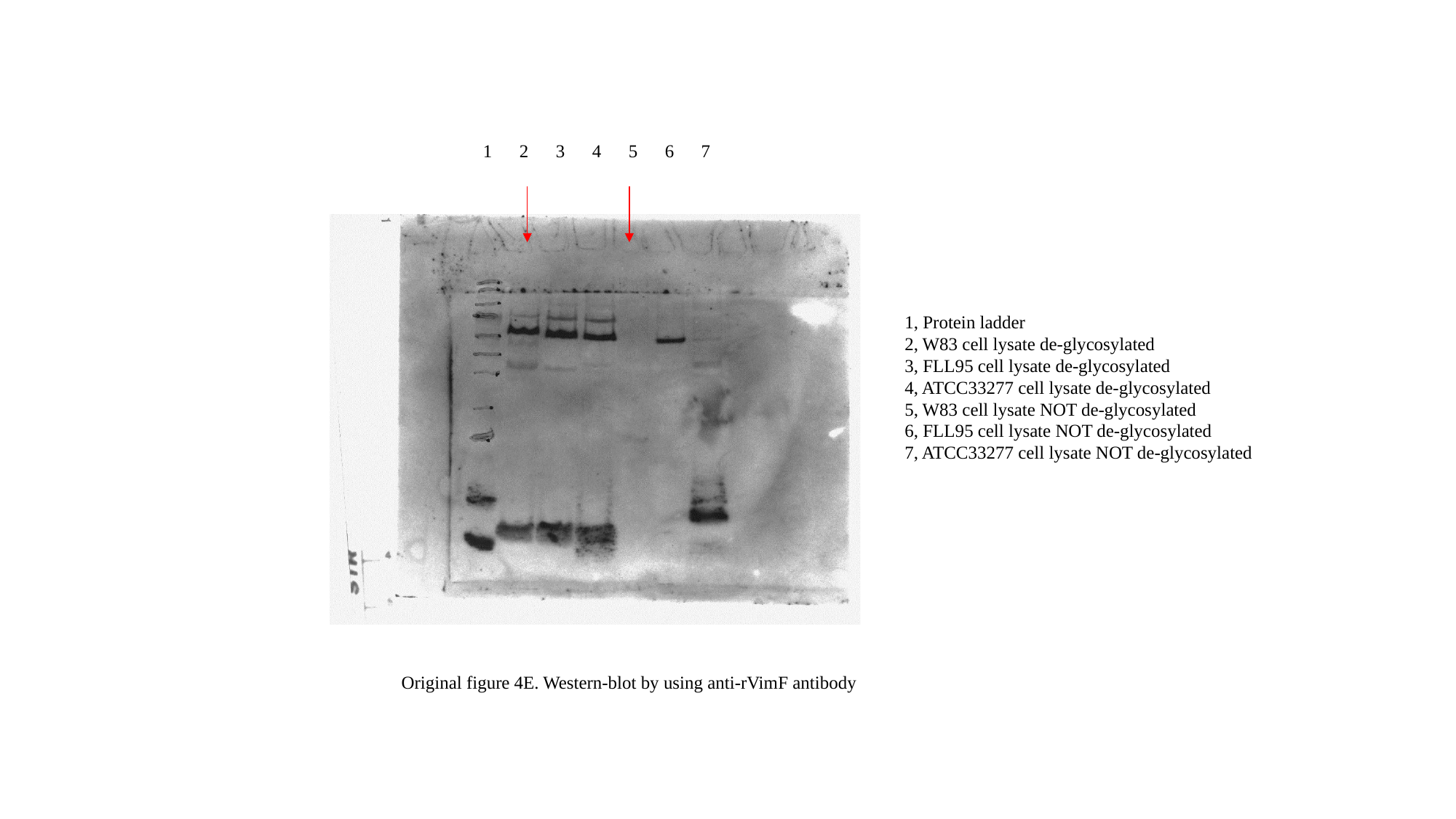

1 2 3 4 5 6 7
1, Protein ladder
2, W83 cell lysate de-glycosylated
3, FLL95 cell lysate de-glycosylated
4, ATCC33277 cell lysate de-glycosylated
5, W83 cell lysate NOT de-glycosylated
6, FLL95 cell lysate NOT de-glycosylated
7, ATCC33277 cell lysate NOT de-glycosylated
Original figure 4E. Western-blot by using anti-rVimF antibody

## Slide 3
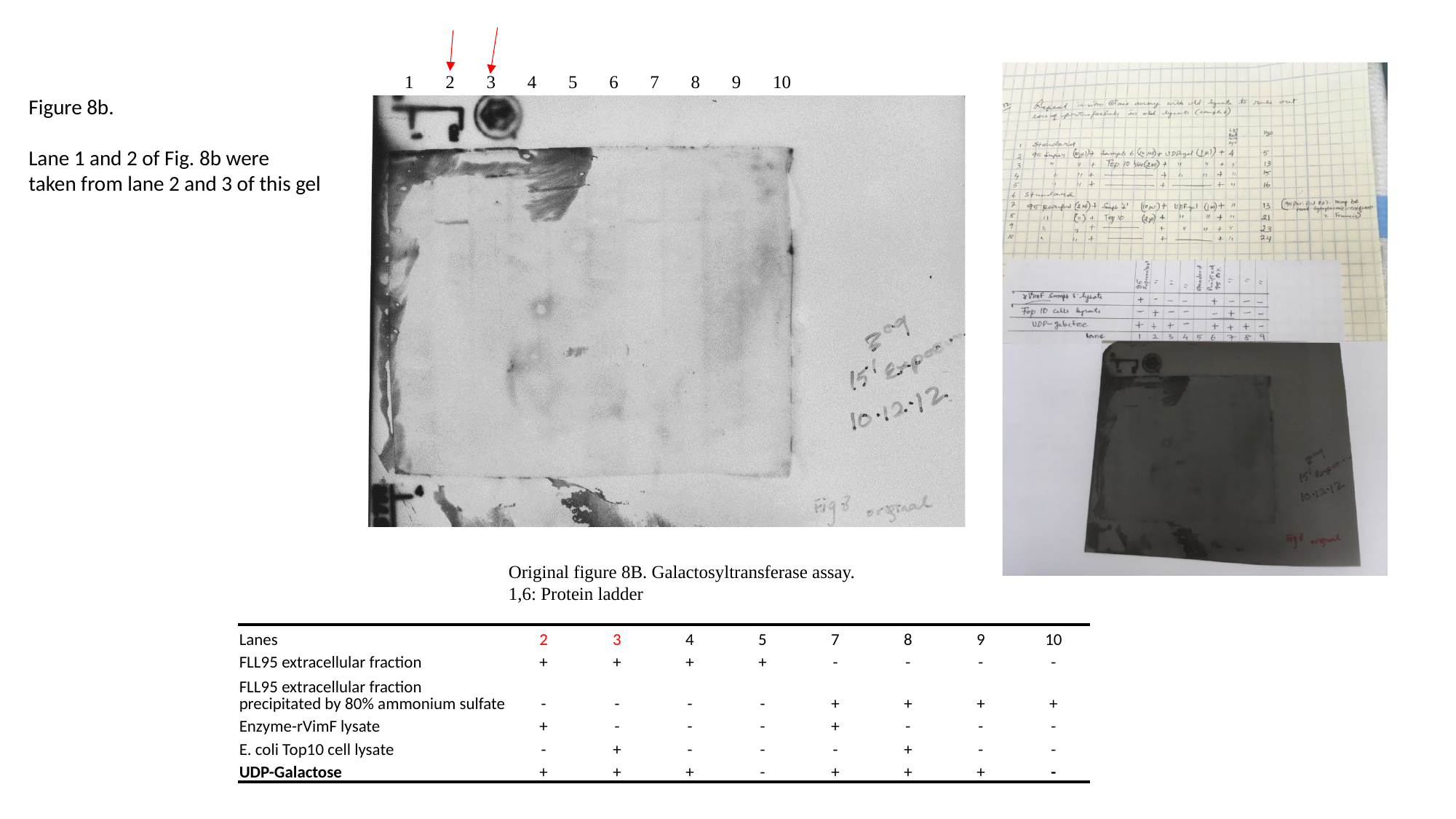

1 2 3 4 5 6 7 8 9 10
Figure 8b.
Lane 1 and 2 of Fig. 8b were taken from lane 2 and 3 of this gel
Original figure 8B. Galactosyltransferase assay.
1,6: Protein ladder
| Lanes | 2 | 3 | 4 | 5 | 7 | 8 | 9 | 10 |
| --- | --- | --- | --- | --- | --- | --- | --- | --- |
| FLL95 extracellular fraction | + | + | + | + | - | - | - | - |
| FLL95 extracellular fraction precipitated by 80% ammonium sulfate | - | - | - | - | + | + | + | + |
| Enzyme-rVimF lysate | + | - | - | - | + | - | - | - |
| E. coli Top10 cell lysate | - | + | - | - | - | + | - | - |
| UDP-Galactose | + | + | + | - | + | + | + | - |

## Slide 4
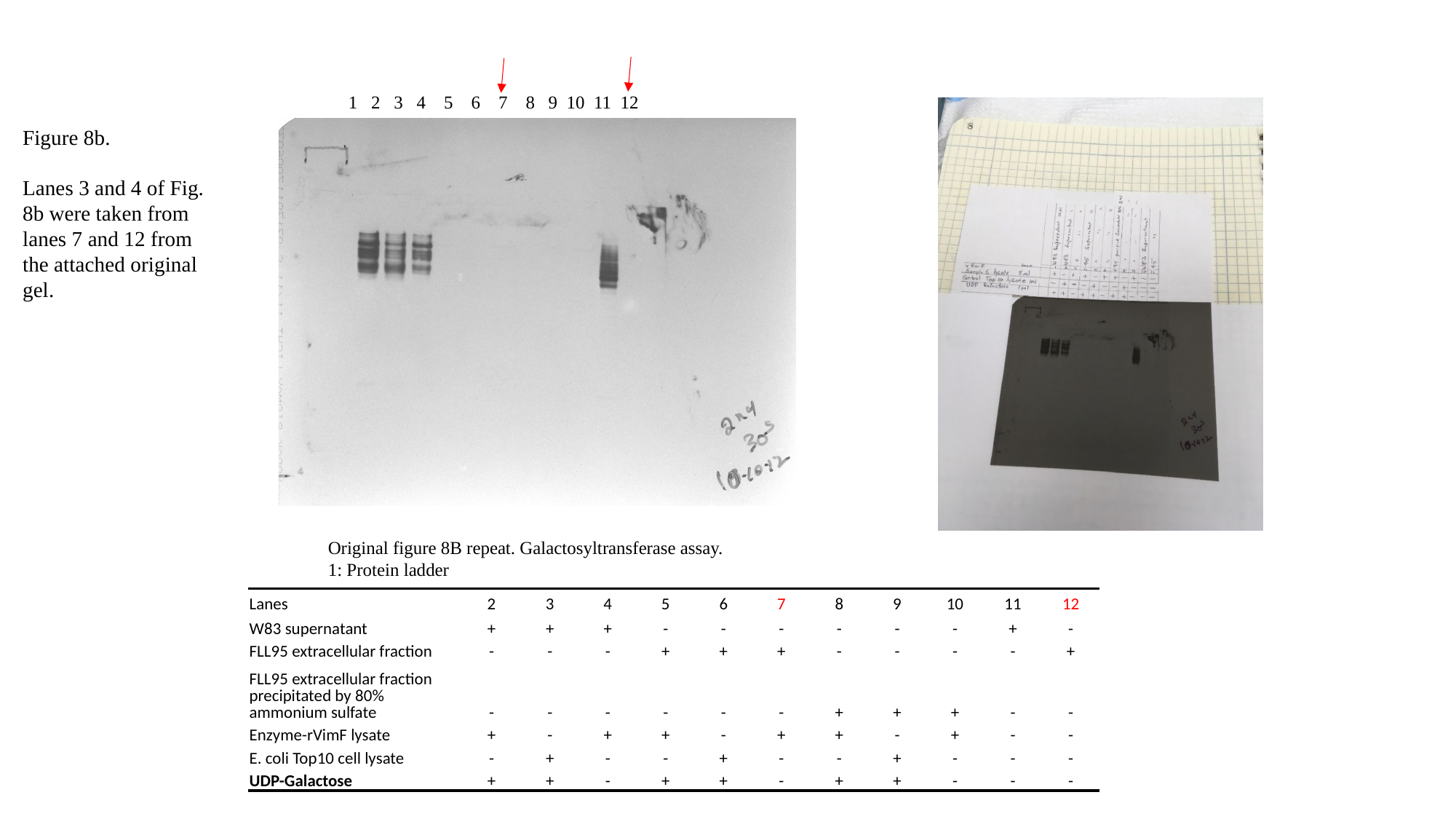

1 2 3 4 5 6 7 8 9 10 11 12
Figure 8b.
Lanes 3 and 4 of Fig. 8b were taken from lanes 7 and 12 from the attached original gel.
Original figure 8B repeat. Galactosyltransferase assay.
1: Protein ladder
| Lanes | 2 | 3 | 4 | 5 | 6 | 7 | 8 | 9 | 10 | 11 | 12 |
| --- | --- | --- | --- | --- | --- | --- | --- | --- | --- | --- | --- |
| W83 supernatant | + | + | + | - | - | - | - | - | - | + | - |
| FLL95 extracellular fraction | - | - | - | + | + | + | - | - | - | - | + |
| FLL95 extracellular fraction precipitated by 80% ammonium sulfate | - | - | - | - | - | - | + | + | + | - | - |
| Enzyme-rVimF lysate | + | - | + | + | - | + | + | - | + | - | - |
| E. coli Top10 cell lysate | - | + | - | - | + | - | - | + | - | - | - |
| UDP-Galactose | + | + | - | + | + | - | + | + | - | - | - |

## Slide 5
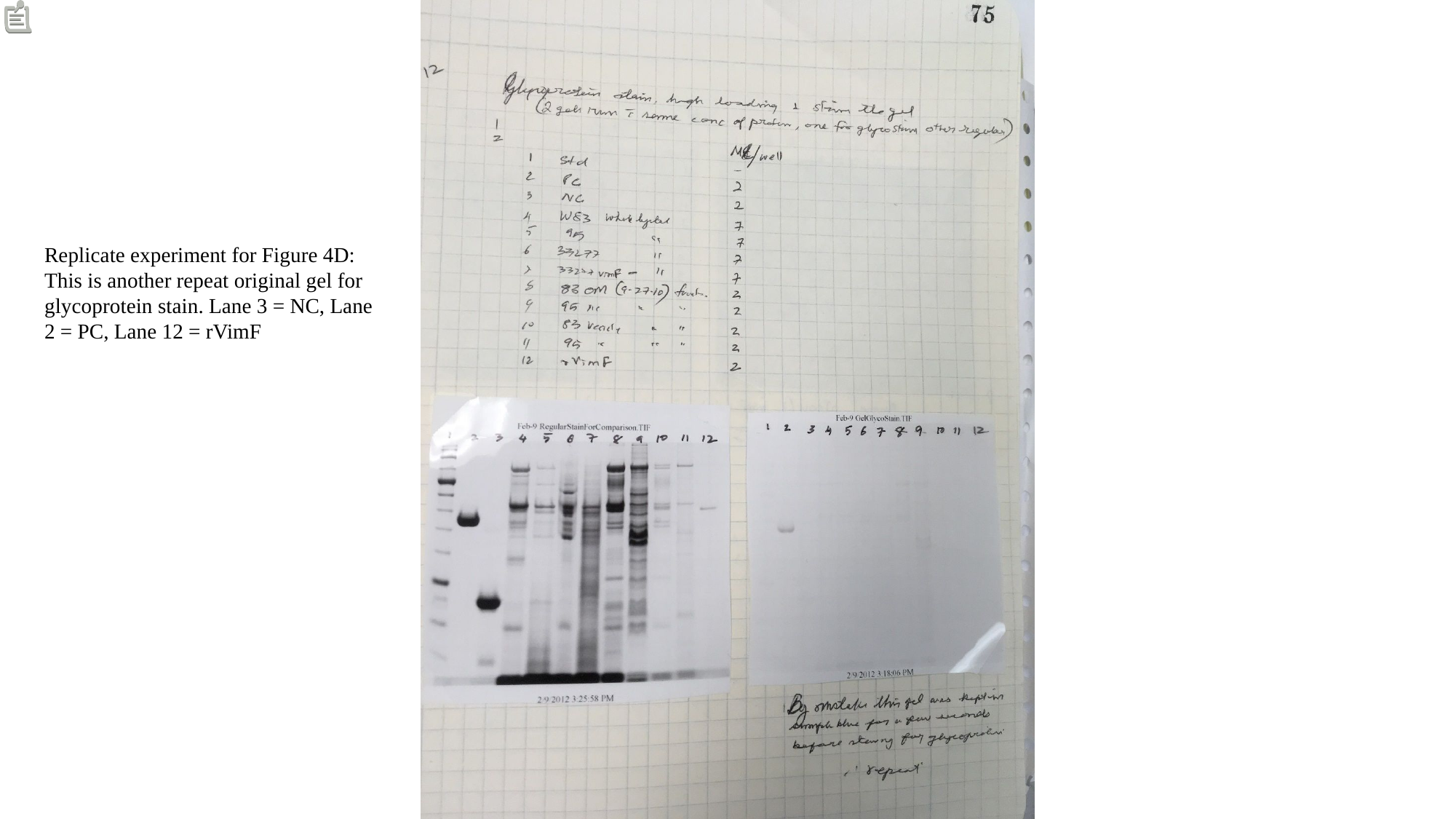

Replicate experiment for Figure 4D:
This is another repeat original gel for glycoprotein stain. Lane 3 = NC, Lane 2 = PC, Lane 12 = rVimF
